# A new electrolyte for molten carbonate decarbonization

**DOI:** 10.1038/s42004-024-01306-z

**Published:** 2024-09-18

**Authors:** Gad Licht, Kyle Hofstetter, Xirui Wang, Stuart Licht

**Affiliations:** 1 C2CNT LLC, A4 188 Triple Diamond Blvd, Venice, FL 34275 USA; 2 Carbon Corp, 1035 26 St NE, Calgary, AB T2A 6K8 Canada; 3 Department of Chemistry, George Washington University, Washington, DC 20052 USA

**Keywords:** Sustainability, Carbon capture and storage, Materials chemistry, Electrochemistry

## Abstract

The molten Li_2_CO_3_ transformation of CO_2_ to oxygen and graphene nanocarbons (GNCs), such as carbon nanotubes, is a large scale process of CO_2_ removal to mitigate climate change. Sustainability benefits include the stability and storage of the products, and the GNC product value is an incentive for carbon removal. However, high Li_2_CO_3_ cost and its competitive use as the primary raw material for EV batteries are obstacles. Common alternative alkali or alkali earth carbonates are ineffective substitutes due to impure GNC products or high energy limitations. A new decarbonization chemistry utilizing a majority of SrCO_3_ is investigated. SrCO_3_ is much more abundant, and an order of magnitude less expensive, than Li_2_CO_3_. The equivalent affinities of SrCO_3_ and Li_2_CO_3_ for absorbing and releasing CO_2_ are demonstrated to be comparable, and are unlike all the other alkali and alkali earth carbonates. The temperature domain in which the CO_2_ transformation to GNCs can be effective is <800 °C. Although the solidus temperature of SrCO_3_ is 1494 °C, it is remarkably soluble in Li_2_CO_3_ at temperatures less than 800 °C, and the electrolysis energy is low. High purity CNTs are synthesized from CO_2_ respectively in SrCO_3_ based electrolytes containing 30% or less Li_2_CO_3_.

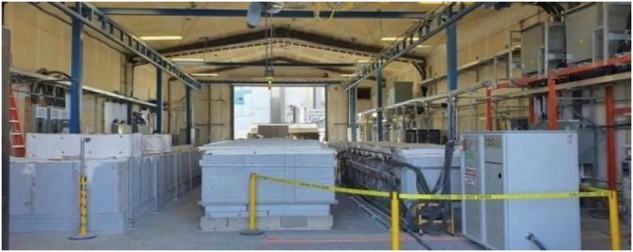

## Abstract (web summary)

The transformation of CO_2_ to oxygen and graphene nanocarbons using lithium carbonate as an electrolyte is a promising, large-scale process for CO_2_ removal and valorization, but lithium carbonate is already in high demand as an important battery material. Here, the authors report the use of strontium carbonate as an alternative electrolyte in the electrochemical reduction of CO_2_ to carbon nanotubes.

## Introduction

In 2015, a decarbonization technique was introduced for the transition metal nucleated transformation of CO_2_ to nanoallotropes of carbon. This single-step decarbonization process in molten carbonates electrochemically splits CO_2_ into carbon and oxygen via the C2CNT (carbon dioxide to carbon nanomaterial technology) process. Catalyzed by transition metals, such as iron, nickel, and chromium, carbon growth is tuned by variations in the composition of the electrolysis electrode, current density, and temperature, forming high-purity graphene nanocarbons (GNCs), such as carbon nanofibers and nanotubes^1–5^. Sustainability benefits include the stability and storage of the GNC products. Their graphene structure is stable for effective sequestration, and the GNC high product value is an incentive for carbon removal. The current value of GNCs is due to the high strength, conductivity electronic, medical and catalytic properties of graphene allotropes and their open market value of approximately a million $US/tonne^1^.

Alternatively, commercial carbon nanotubes are generally produced by chemical vapor deposition (CVD)^6–10^, often with a large carbon footprint due to chemical fuel precursors and increased energy consumption^11^. The CVD synthesis of carbon nanotubes includes organometallics. A recent study presented the formation of carbon nanofibers from CO_2_ in a multistep process^12^. First, CO_2_ and water were electrolyzed to form syngas, and then, the syngas was used as a reactant to produce carbon nanofibers by CVD. In particular, the study stated that a disadvantage of C2CNT, rather than CVD, is that C2CNT competes for limited lithium carbonate supplies used in the battery industry (for example, lithium carbonate is a principal precursor in the fabrication of Li-ion batteries in EVs).

The physical and chemical systems for carbon nanotube (CNT) synthesis using conventional chemical vapor deposition (CVD) differ significantly from those of the new C2CNT synthesis. CVD is a chemical process that occurs at a gas/solid interface, typically using various organometallic compounds as reactants, and is associated with a high carbon footprint. In contrast, C2CNT is an electrochemical process that transforms CO_2_ into CNTs through molten electrolysis, operating at a liquid/solid interface with a carbon-negative footprint.

The C2CNT process benefits from a molten carbonate electrolyte that provides a higher density of reactive carbon sites—specifically, tetravalent carbon available for reduction at the molten carbonate/cathode interface—compared to the lower density of carbon available as a gas in CVD. While CVD may apply an electric field to the substrate during CNT growth, C2CNT consistently involves a strong electric field that rapidly decreases through the double layer adjacent to the cathode. One of the key advantages of C2CNT is that its production cost is predominantly influenced by the cost of electrons (electricity), leading to substantial cost reductions compared to conventional CVD methods.

**Fig. 1:**
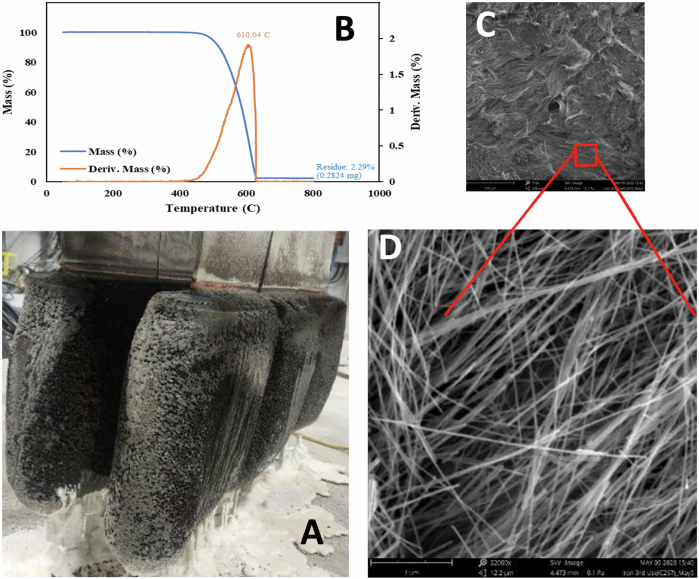
Large-scale C2CNT electrolysis. **A** Photo of an extracted cathode after current density J = 0.2 A/cm^2^ electrolysis of CO_2_ in a pure 750 °C Li_2_CO_3_ electrolyte. **B** TGA, **C**, **D** SEM images of the washed carbon nanotube product. The SEM images are at magnifications of (**B**) 710× and (**C**) 2250×.

The C2CNT electrodes were scaled up by one thousand-fold from the 5 cm^2^ electrodes used in the 2015 design and assembled in electrolysis modules, which collectively comprise 1000 tons of CO_2_ decarbonization. Panel A of Fig. 1 shows a photograph of the scaled-up brass cathode extracted after electrolysis of CO_2_ for 16 h in a lithium carbonate electrolyte. After electrolysis, the product is subsequently pressed and/or washed to remove and recover the remaining electrolyte from the product. SEM images of the washed products were obtained at magnifications of 710× and 2250×, and the images show that the CNTs had a purity >>90%. Panel B of the figure shows the TGA analysis and a product purity >97%. Furthermore, TGA exhibited an inflection temperature of T_infl_ = 610 °C, which is a combustion point consistent with an oxidation-resistant nanographene structure and is unlike an alternative common amorphous carbon that oxidizes at several hundred degrees lower temperature.

Subsequent to this electrolysis, fivefold larger cathodes are regularly used. The average GNC purity ranged from 90 to 98%, depending on the pure Li_2_CO_3_ electrolysis conditions and post-electrolysis press extraction and/or polishing washing. In accordance with the electrolytic splitting of CO_2_: CO_2_ - > C_GNC_ + O_2_, 1 tonne of GNC is synthesized via the C2CNT process, which removes 3.7 tonnes of CO_2_ from the atmosphere or flue gas. GNCs have found applications in materials such as medicine, polymers, batteries, cement, and textiles^13–26^.

Variations in the electrolysis electrode composition, current density, oxide addition, and electrolysis temperature tune C2CNT electrolysis to form long^27,28^, tangled^1,29^, thin-walled^30^, helical^31^, magnetic^32^, nanobamboo, branched, and nanopearl carbon nanotube, and conical nanofiber morphologies^4,5^. The addition of boron, sulfur, or nitrate salts produces doped carbon nanotubes^27,28,33^. Further variations in the lithium carbonate electrolysis conditions facilitate the formation of alternative, pure carbon nanoallotropes, including solid or hollow carbon nanoallotropes^4,34^, graphene nanoscaffolds^35^, graphene nanoplatelets, or graphene^36^.

Li_2_CO_3_ is expensive, and this is in part due to the competitive demand for Li_2_CO_3_, particularly for use in the preparation of Li-ion batteries for the growing electronic vehicle (EV) market. Global Li_2_CO_3_ prices for 2022 to 2024 vacillate in the range of $10,000–75,000 per tonne. These elevated prices present a cost constraint to the alternative use of Li_2_CO_3_ as a molten electrolyte in the transformation, by electrolytic splitting, of CO_2_ in Li_2_CO_3_ to GNCs. This study develops the fundamental chemistry and demonstrates the efficacy of a new, substantially more cost-effective electrolyte for molten carbonate decomposition.

We had explored the growth of CNTs in alternative molten carbonate electrolytes, often without success. The pure salts Li_2_CO_3_, Na_2_CO_3_, and K_2_CO_3_ have melting points at 723 °C, 851 °C and 891 °C, respectively. Eutectic ternary mixes of Li_2_CO_3_, Na_2_CO_3_, and K_2_CO_3_ have been well characterized as molten carbonate electrolytes and do not produce significant amounts of CNT products^37^. Potassium carbonate, as a component of a binary lithium carbonate electrolyte, tends to disrupt the electrocatalytic, highly stable oxide layer that forms on the electrolysis anode and results in corrosion of the anode^38^. Increasing K_2_CO_3_ also inhibits transition metal nucleation at higher concentrations^39^. The synthesized carbon nanotubes are increasingly defect-ridden at contents of 20% K_2_CO_3_ or higher^39^. At 50 wt% K_2_CO_3_ in Li_2_CO_3_, metallic potassium, rather than carbon, forms, and the product ignites when exposed to humid air, while no CNTs are formed from a Na_2_CO_3_-K_2_CO_3_ electrolyte^37,38^. Upon electrolysis, a binary mixture of sodium and Li_2_CO_3_ produces CNTs up to 20 wt% Na_2_CO_3_, but beyond that, the product is increasingly deformed^39^. Interestingly, at a lower electrolysis temperature of 670 °C, rather than 770 °C, 50 wt% sodium carbonate and 50 wt% Li_2_CO_3_ form another GNC, other than CNTs, which we have termed carbon nanoscaffolds^35^. Furthermore, the addition of Na_2_CO_3_ to Li_2_CO_3_ considerably increases the electrolysis potential^2^.

Magnesium carbonate decomposes to magnesium oxide and CO_2_ above 350 °C, while calcium carbonate decomposition to lime above 840 °C is the basis of cement production. Barium carbonate melts at 811 °C and has a eutectic morphology with lithium carbonate at 609 °C^40^. The addition of magnesium carbonate to lithium carbonate suppresses CNT formation, resulting in a product with a honeycomb morphology with only a small amount of thin-walled CNTs^33^. CNTs are grown in lithium carbonate containing up to approximately 20 wt% calcium and barium carbonate^33,37,41,42^. CO_2_ electrolysis in a mixed calcium carbonate/lithium carbonate electrolyte proceeds differently than that in other mixed lithium carbonate electrolytes. In lithium or lithium/barium electrolytes, lithium or barium oxide is highly soluble, whereas calcium oxide is soluble only to 0.2 m CaO in Li_2_CO_3_^40,41^. Hence, during electrolysis, rather than reacting with CO_2_, the oxide precipitates out as calcium oxide, while calcium carbonate is consumed rather than CO_2_ splitting. The addition of magnesium, calcium, or barium carbonate to lithium carbonate was observed to cause an unfavorable increase in the electrolysis potential^2,33^.

Despite its high solidus temperature of 1494 °C, in the present study, strontium carbonate was shown to be unusually soluble in lithium carbonate at temperatures less than 800 °C. Strontium carbonate is the only carbonate with a similar thermodynamic affinity for CO_2_ to that of lithium carbonate, and as with lithium carbonate, it supports low-energy decarbonization to form useful CNT products. Concentrated strontium carbonate electrolytes are demonstrated here to form high-purity CNT products, and as opposed to lithium carbonate are a cost-effective electrolyte for molten carbonate electrolysis. To date, there have been no successful decarbonization chemistries deployed to meaningfully mitigate planetary climate change. Climate change is an existential threat to the planet, and to the majority of the species on the planet including humankind. The new strontium decarbonization chemistry presented in this study has the potential to be the first such decarbonization chemistry. The never before described strontium chemistry is analyzed in depth from a thermodynamic and practical standpoint.

## Results and discussion

### Electrolysis and electrolysis potentials in molten carbonate

An Illustration of the C2CNT process, detailed SEM, TEM, HAADF, RXRD and Raman of the synthesized carbon nanotubes, as well as examples of the range of graphene nanocarbon allotropes synthesized from CO_2_ by molten carbonate electrolysis are included in the Supplementary Material.

The electrochemical reduction of CO_3_^2−^ in molten carbonate is a 4e^-^ process:

1 $${{{{\rm{CO}}}}_{3}}^{2-}({{\rm{molten}}})\to {{\rm{C}}}({{\rm{nanomaterial}}})+{{{\rm{O}}}}_{2}({{\rm{gas}}})+{{{\rm{O}}}}^{2-}({{\rm{dissolved}}})$$

The CO_2_ added to the electrolyte chemically reacts with the oxide formed through Eq. 1 to renew CO_3_^2−^ following Eq. 2:

2 $${{{\rm{CO}}}}_{2}({{\rm{gas}}})+{{{\rm{O}}}}^{2-}({{\rm{dissolved}}})\to {{{{\rm{CO}}}}_{3}}^{2-}({{\rm{molten}}})$$

Combining Eqs. 1 and 2 yields a net decarbonization reaction:

3 $${{{\rm{CO}}}}_{2}({{\rm{gas}}})\to {{\rm{C}}}({{\rm{nanomaterial}}})+{{{\rm{O}}}}_{2}({{\rm{gas}}})$$

**Fig. 2:**
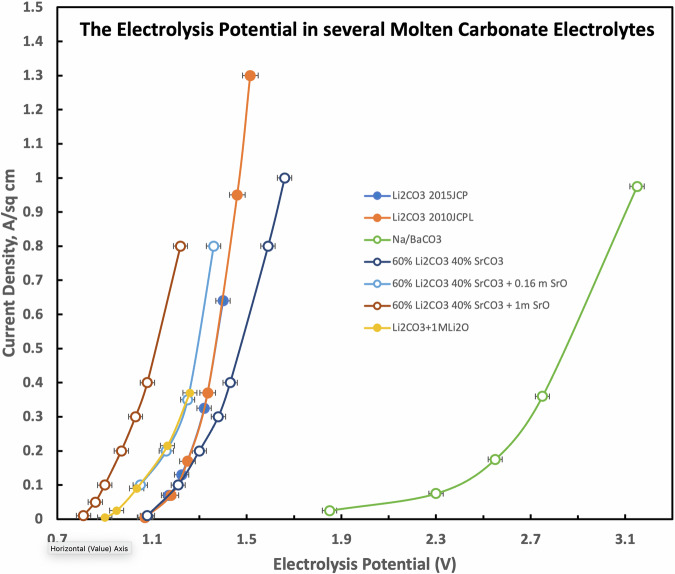
Carbonate electrolysis potential measured in several molten carbonates. The electrolytes investigated included pure lithium carbonate or pure lithium carbonate with 1.0 m Li_2_O, an equal mixture of sodium and barium carbonate, a mixture of 40 wt% strontium carbonate with 60 wt% Li_2_CO_3_, or a mixture of the latter with either 0.16 or 1.0 m SrO and are reproducible to 30 mV. Electrolysis Potentials of Li_2_CO_3_ with or without Li_2_O was obtained from our measurements in refs. ^2,56^, and Na/BaCO_3_ was obtained from ref. ^2^. The additional strontium-containing electrolyte electrolysis potentials were measured in this study.

We previously synthesized CNTs by electrolysis in 50/50 wt% Na/BaCO_3_, albeit by forming CNTs at a lower purity than that of pure Li_2_CO_3_^43^. However, this synthesis requires severalfold more electrolysis power to drive the reaction. Figure 2 compares the molten carbonate electrolysis potential of several electrolytes. Compared to 1 V, which drives CNT formation in lithium carbonate, the Na/BaCO_3_ potential results in a two- to threefold greater voltage and inordinately high energy consumption to drive a decarbonization process.

### The unexpectedly high solubility of strontium salts in molten Li_2_CO_3_

Interestingly, there is little, or no, information available on the melting point of the binary mixture of pure Li_2_CO_3_/(mp 723 °C) with SrCO_3_ (which is solid to 1494 °C). One study revealed that SrCO_3_ fully decomposes to SrO as the temperature increases from 875 °C to 1035 °C^44^. It is discovered that SrCO_3_ is highly soluble in molten lithium carbonate at temperatures <800 °C and that the inexpensive SrCO_3_ salt can replace a major portion of the expensive lithium carbonate salt as an electrolyte for decarbonization and CNT growth. We find that strontium oxide, SrO, which can facilitate the rapid reactive dissolution of CO_2_, is also highly soluble (at ~25 wt% in Li_2_CO_3_ at 750 °C) when measured here using 99% SrO. The efficacy of these salts as electrolytes for molten electrolysis at temperatures below 800 °C is important because above these temperatures, CO_2_ increasingly electrolytically splits to gaseous carbon monoxide rather than to the desired solid-phase GNC products, and by 950 °C, the product is entirely CO rather than solid GNCs^43^.

**Fig. 3:**
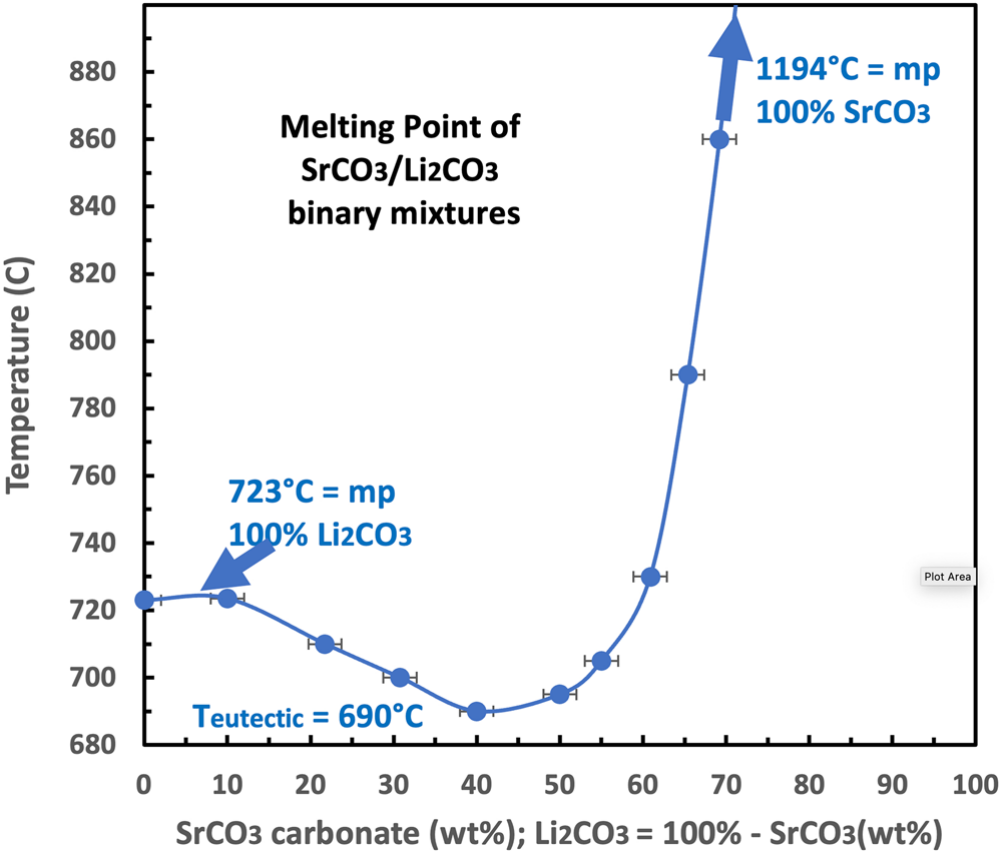
The melting point and high SrCO_3_ solubility of binary mixtures of SrCO_3_ and Li_2_CO_3_. Measured solubilities are reproducible to <2%.

The measured melting points of binary mixtures of SrCO_3_ and Li_2_CO_3_ as a function of temperature are presented in Fig. 3. The figure shows that the solubility of 99.4% purity SrCO_3_ in Li_2_CO_3_ (99.8%, Green Chemical Co.) reaches 65 wt% in the regular CO_2_ splitting temperature domain (T < 800 °C). The binary mixture exhibited a minimum (eutectic point) melting point at 690 °C occurring at a composition of 40 wt% SrCO_3_. It is likely that ternary materials composed of Li-SrCO3 plus other salt mixes will exhibit lower eutectic temperatures. A lower 98.6% purity SrCO_3_, containing minor ternary mix components (0.8% BaCO_3_ and 0.2 wt% CaCO_3_), exhibited a slightly lower eutectic temperature of 880 °C at 40 wt% composition in the same 60 wt% Li_2_CO_3_.

SrCO_3_ is available at a more stable global price of approximately $1,040/ton, a cost that is 1 to 2 orders of magnitude less than that of Li_2_CO_3_^45,46^. Strontium is the tenth most abundant metal in the earth’s core, while lithium is twenty-fourth most abundant^47^. Strontium carbonate is widely mined and refined to from strontium sulfate or carbonate. Previously, strontium carbonate was used in glass compositions for television and cathode ray devices but is not used in today’s flat screens. Today, strontium is used in pyrotechnics and in various applications, including ceramics, ferrite magnets, superconductors, biomaterials, chemical sensors, and catalysts^48–50^. It is also used to protect certain magnesium alloys against corrosion^51,52^ and in specialized cement compositions^53–55^. However, SrCO_3_ is solid at 1494 °C, which is too high for CO_2_ electrolysis to solid carbon products. We have demonstrated that temperatures below 800 °C are suitable for CO_2_ molten carbonate electrolysis. At higher temperatures, another product, carbon monoxide, increasingly forms, and the product is pure carbon monoxide at 950 °C^56^. Carbon monoxide is not preferred as a decomposition product. Its main use is as an oxidant, and in that process, it returns CO_2_ to the atmosphere. Alternatively, GNCs retain the high geologic stability of mineral graphite to sequester CO_2_.

### The overlapping affinity of strontium and lithium carbonate for binding and releasing CO_2_

CO_2_ is a critical decarbonization component in molten carbonate electrolytic splitting and the transformation of CO_2_ to GNCs. In particular, the affinity of lithium carbon for CO_2_ provides a balance both facilitating rapid CO_2_ intake into the molten salt and providing an enriched carbon electrolytic media. The enriched carbon electrolytic media facilitates the electrochemical reduction of tetravalent carbon to carbon. The enriched media has an observed the low overpotential to generate high electrolysis rates and also specifically generates GNCs, as observed by the high purity of GNCs, such as CNTs. Here, we calculate the CO_2_ affinity of SrCO_3_ and show that, unlike nonlithium alkali carbonates or other alkali earth carbonates, SrCO_3_ exhibits a CO_2_ affinity equivalent to that of Li_2_CO_3_. The equilibrium, K(MCO_3_), for alkali and alkali earth carbonates to separate into CO_2_ and oxide, or to form carbonate from them in the reverse reaction, is given by the equilibrium for the decomposition, or in reverse for formation, of a carbonate from CO_2_ and its oxide:

4 $${{\rm{M}}}{{\rm{CO}}}{{\scriptstyle{3}}} \, {\rightleftharpoons} \, {{\rm{CO}}}{{\scriptstyle{2}}}+{{\rm{MO}}} \quad \quad \quad \quad \quad {{\rm{K}}}{{\rm{\scriptstyle{MCO}}}}{\scriptstyle{3}} = {{\rm{p}}}{{\rm{co}}}{\scriptstyle{2}} \, {{{\rm{a{\scriptstyle{MO}}}}}}/{{\rm{a{\scriptstyle{M}}}}}{{\rm{\scriptstyle{CO}}}}{\scriptstyle{3}}$$

where examples of M are Li_2_, Na_2_, K_2_, Mg, Ca, Ba or Sr.

The equilibrium, K_MCO3_, is calculated from the relationship between the equilibrium and free energy, where the gas constant R = 8.31 J/mol K:

5 $$\Delta {{\rm{G}}}^{\circ} \, {{{\rm{M}}}}{{\rm{\scriptstyle{CO}}}}{\scriptstyle{3}} = -{{{\rm{RTlnK}}}}{{\rm{\scriptstyle{MCO}}}}{\scriptstyle{3}}; \quad {{{\rm{K}}}}{{\rm{\scriptstyle{MCO}}}}{\scriptstyle{3}} \, ({{\rm{T}}}) = {{{\rm{e}}}}^{-\Delta {{\rm{G}}}({{\rm{T}}})/{{\rm{RT}}}}$$

K_MCO3_ is calculated from the thermochemical free energies for a variety of alkali and alkali earth carbonates, their oxides, and CO_2_.

**Fig. 4:**
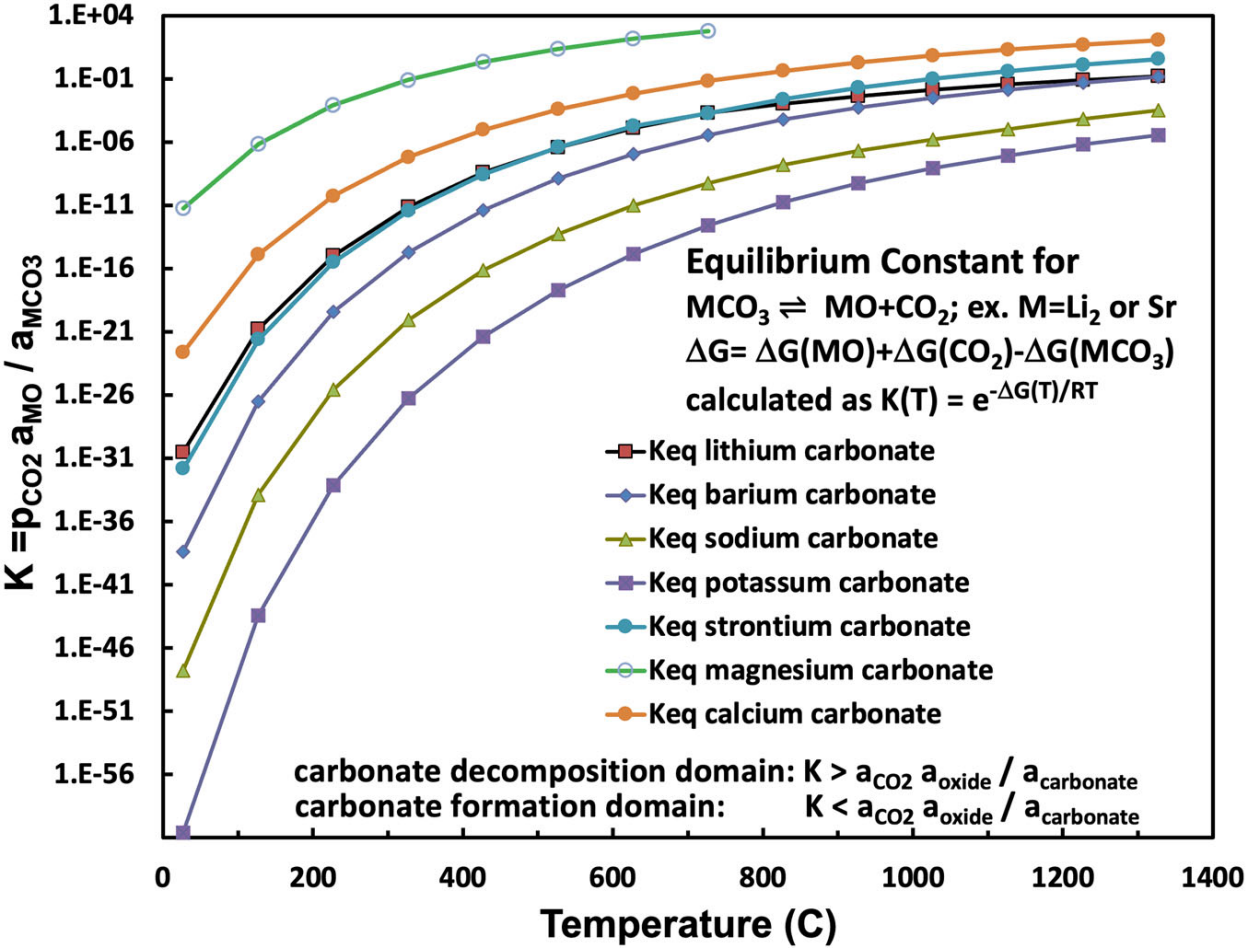
Equilibrium constant for CO_2_ release for a range of alkali and alkali earth carbonates. The equilibrium constants as a function of temperature for strontium, lithium, sodium, potassium, and barium carbonate. The equilibrium constants are calculated from the free energy according to Eq. 5. The free energy is calculated from the metal carbonate, metal oxide, and carbon dioxide enthalpies and entropies^2,69–72^.

Figure 4 presents a comparison of carbonate equilibrium constants for binding and releasing carbon dioxide by strontium carbonate compared to those for alkali or other alkali earth carbonates. These values are plotted as a function of temperature. Above any given salt equilibrium curve, that is, in the low CO_2_ activity domain (a_CO2_ a_oxide_/a_carbonate_ < K), the salt will spontaneously decompose, while in the high CO_2_ activity domain, the salt will spontaneously form from CO_2_ and the salt’s oxide. Interestingly, as shown in the figure, the strontium carbonate equilibrium is similar to that of lithium carbonate and very different from that of the other carbonate salts. Specifically, the carbonate equilibrium constants for strontium and lithium carbonate are nearly identical in the 400 °C to 800 °C range, in which lithium carbonate binary and trinary salt mixes are molten. We previously observed a high tendency for electrolytic graphene nanocarbon formation in the 600 °C to 800 °C temperature range. At lower temperatures, transition metal nucleation growth of carbon /nanotubes is not observed^37^, and at increasing temperatures above 800 °C, 2-electron reduction to CO, rather than 4-electron reduction to carbon, increasingly dominates. The comparable nature of strontium to lithium carbonate equilibria provides an unusual environmental media conducive to the electrolytic splitting of carbon dioxide and its transformation to graphene nanocarbons.

Isotopic tracking with ^13^C was employed to follow the reduction of CO_2_ as it is dissolved in molten carbonate and undergoes electrolytic splitting to generate the building blocks of CNTs^3^. Mechanistically in the 600° to 800 °C domain maximizing CNT yield and purity, we hypothesize that the facilitated molten carbonate growth is related to a thermodynamic “Goldilocks” range with an optimal activation barrier for the kinetic binding and reduction of CO_2_ in accord with Eqs. 2, 3 and 4. More specifically, at 750 °C, in accord with Fig. 4, K_MCO3_, the equilibrium constant for CO_2_ release calculated from the Eq. 5 free energies of the carbonate and oxide salts and CO_2_, are 700 and 0.1 respectively for magnesium and calcium carbonate. In these cases, CO_2_ is weakly bound. This is consistent with the low temperature of calcination decomposition of these salts. This facile release of CO_2_ to the gas phase provides an insufficient source in the molten salt for CO_2_ splitting inhibiting CNT growth. Conversely at 750 °C K_MCO3_ are 6 × 10^−6^, 1 × 10^−9^, and 5 × 10^−13^ respectively for barium, sodium and potassium carbonate indicative in each case that CO_2_ is tightly bound (favoring the left side of Eq. 4) and less available for CO_2_ splitting in the 600° to 800 °C range.

As opposed to the too loosely bound CO_2_ in magnesium and calcium carbonate, and the too tightly bound CO_2_ in barium, sodium, and potassium carbonates, the “Goldilocks” CO_2_ carbonate binding for 750 °C K_MCO3_ is calculated as 3 × 10^−4^ and 7 × 10^−4^ respectively for lithium and strontium carbonate. From the high-quality syntheses from strontium carbonate-based electrolytes which will be presented in the remainder of this study, it may be that the somewhat stronger CO_2_ binding by strontium carbonate may be closer to the ideal K_MCO3_ than that of lithium carbonate.

### The overlapping electrolysis potentials of strontium and lithium carbonate

Figure 2 compares the electrolysis potentials measured in 40 wt% strontium carbonate/60 wt% lithium carbonate electrolytes, both with and without added oxide, and compares these potentials to the electrolysis potentials for pure Li_2_CO_3_ with and without oxide and for the Na/BaCO_3_ electrolyte. As shown in the figure, the electrolysis potential in pure Li_2_CO_3_, as indicated by the solid orange and dark blue dots, decreases when 1 m of Li_2_O is added to the electrolyte, as indicated by the solid yellow dots. The observed onset potential for CO_2_ reduction decreases from 1.08 V in the pure Li_2_CO_3_ electrolyte to 0.9 V with 1 m of Li_2_O. As shown in the figure, the onset potential is the same for pure Li_2_O_3_ as for the 40%/60% SrCO_3_/Li_2_CO_3_ electrolyte. This finding correlates with the similarity discussed above in the equilibrium constants for the two salts in Fig. 3. The 40%/60% SrCO_3_/Li_2_CO_3_ 770 °C electrolysis potentials are presented as hollow dark blue circles. This electrolyte exhibited a moderately higher overpotential at increasing current density than did pure Li_2_CO_3_. Interestingly, the 40%/60% mixed electrolyte is more sensitive to oxide addition when SrO is added than is the pure Li_2_CO_3_ electrolyte when Li_2_O is added. As shown in the figure, the addition of only 0.16 m SrO to the mixed electrolyte results in a similar decrease in potential to that in the 1 m Li_2_O Li_2_CO_3_ electrolyte. As seen by the brown circles in the figure, the addition of 1 m SrO to the 40%/60% SrCO_3_/Li_2_CO_3_ electrolyte further decreases the electrolysis potential to an onset potential of only 0.8 V, and even at higher current densities, the electrolysis potential is lower than that in the Li_2_CO_3_ electrolytes.

### Concentrated strontium electrolyses at 0.2 A/cm^2^ or 0.6 high A/cm^2^ current density

**Fig. 5:**
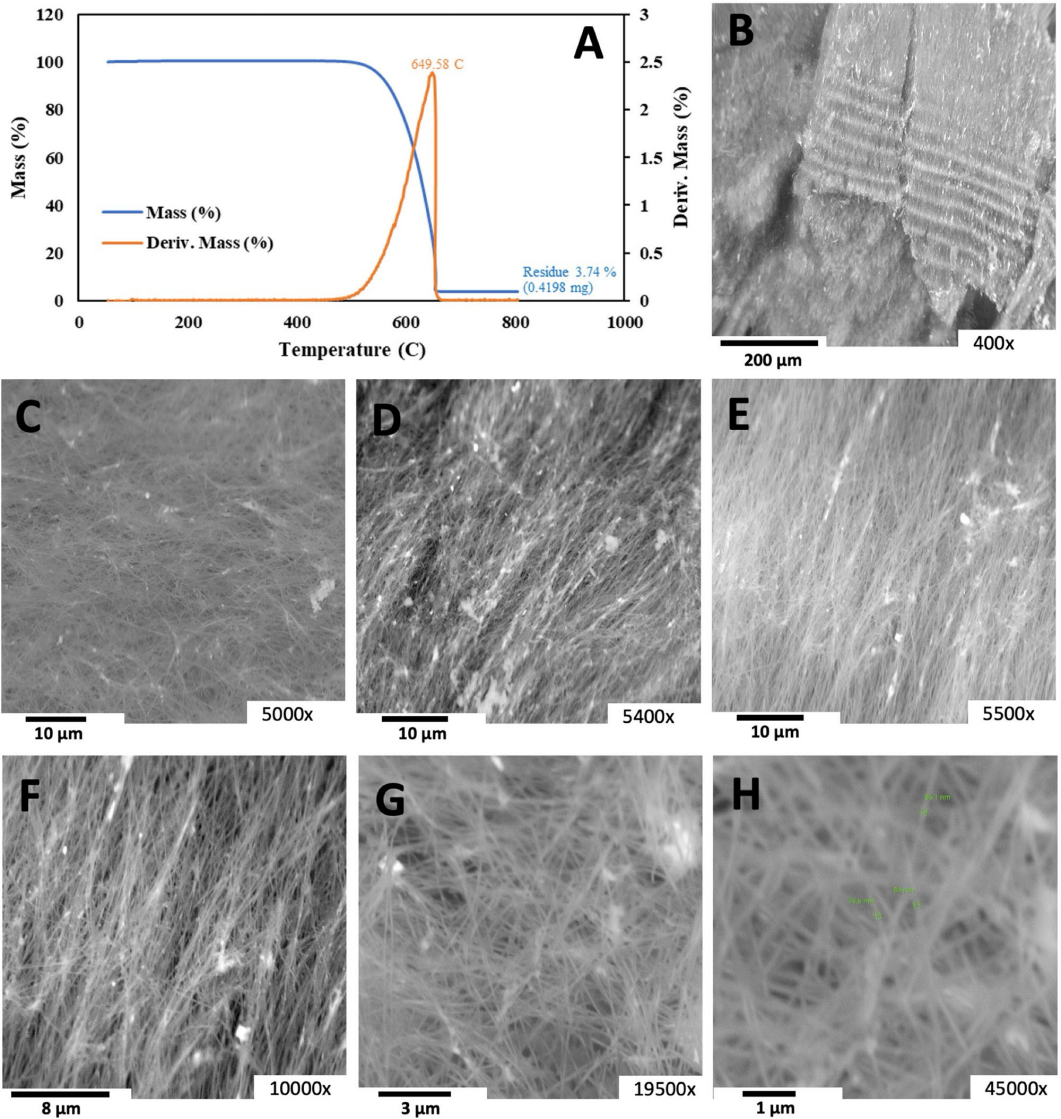
TGA and SEM images of the carbon nanotube product of 25 wt% SrCO_3_ in a Li_2_CO_3_ electrolyte. Four-hour electrolysis was conducted at 750 °C and J = 0.2 A/cm^2^ with a stainless steel 304 anode at a 65 cm^2^ area brass cathode. **A** TGA. The SEM magnifications are as follows: **B** 400×, **C** 5000×, **D** 5400×, **E** 5500×, **F** 10000×, **G** 19500×, **H** 45,000× magnification.

Electrolyzing was performed at 750 °C in lithium media with increasing concentrations of strontium carbonate using a vertical, flat Muntz brass cathode sandwiched between vertical, flat stainless steel cathodes (the anodes are walls of the carbon pot). Electrolysis was studied as a function of electrolyte composition, electrolysis current density, electrolysis time, number of repeated uses of the electrolyte and carbon pot, and electrolysis electrode size. For electrolytes containing 10, 25, 35, or 45% strontium carbonate at 750 °C, the resultant high-purity CNT product was comparable to that obtained with a pure lithium carbonate electrolyte. Figure 5 shows the TGA and SEM results for the product obtained from electrolysis of 25 wt% SrCO_3_ in Li_2_CO_3_ at a current density of J = 0.2 A/cm^2^ for 4 h. As seen via SEM, compared with those of the pure Li_2_CO_3_ product, the CNTs are of comparable high (» 90%) purity (Fig. 1), and according to the TGA results, the post-combustion residue is less than 4%, while the TGA inflection point temperature for combustion is 650 °C. EDS along the CNT strands under an SEM revealed 100% elemental carbon, while the SEM bright spots at the CNT tips were iron^5^. In prior studies, we have extensively documented the Raman spectra, TEM results, points of nucleation, EDS and HAADF elemental analysis, and X-ray diffraction data of synthesized GNCs^1–5,27–39^. In this study, we focus instead on the physical chemistry of the solubility, equilibration, and demonstration of the synthesis of high-purity GNCs, such as CNTs and carbon nano-onions, with an unusual series of readily available strontium carbonate electrolytes to ensure their wide availability for large-scale decarbonization.

A comparable CNT product was obtained at an electrolysis current density of 0.2 A/cm^2^ for both less concentrated (10 wt%) and more concentrated (35 wt%) SrCO_3_ in Li_2_CO_3_ electrolytes, each studied at a current density of J = 0.2 A/cm^2^. Additional electrolysis in the 25 wt% SrCO_3_ electrolyte was repeated a total of 9 times, reusing the same electrolyte and the same carbon pot and cathode. Electrolysis yielded comparable quality CNT products with no indication of deterioration of the carbon pot, the anode comprising the inner walls of the carbon pot or the cathode.

In addition to a 4-h electrolysis at a current density of 0.2 A cm^−2^, electrolyses were also conducted in a 25 wt% SrCO_3_ electrolyte at a lower current density (0.1 A cm^−2^) and for a longer electrolysis duration (16 h). Finally, electrolysis at a high current density of 0.4 A cm^−2^ for four hours was performed in the 35 wt% SrCO_3_ electrolyte. In both cases, the 0.1 A and 0.4 A electrolyses again produced a comparable quality of carbon nanotubes.

**Fig. 6:**
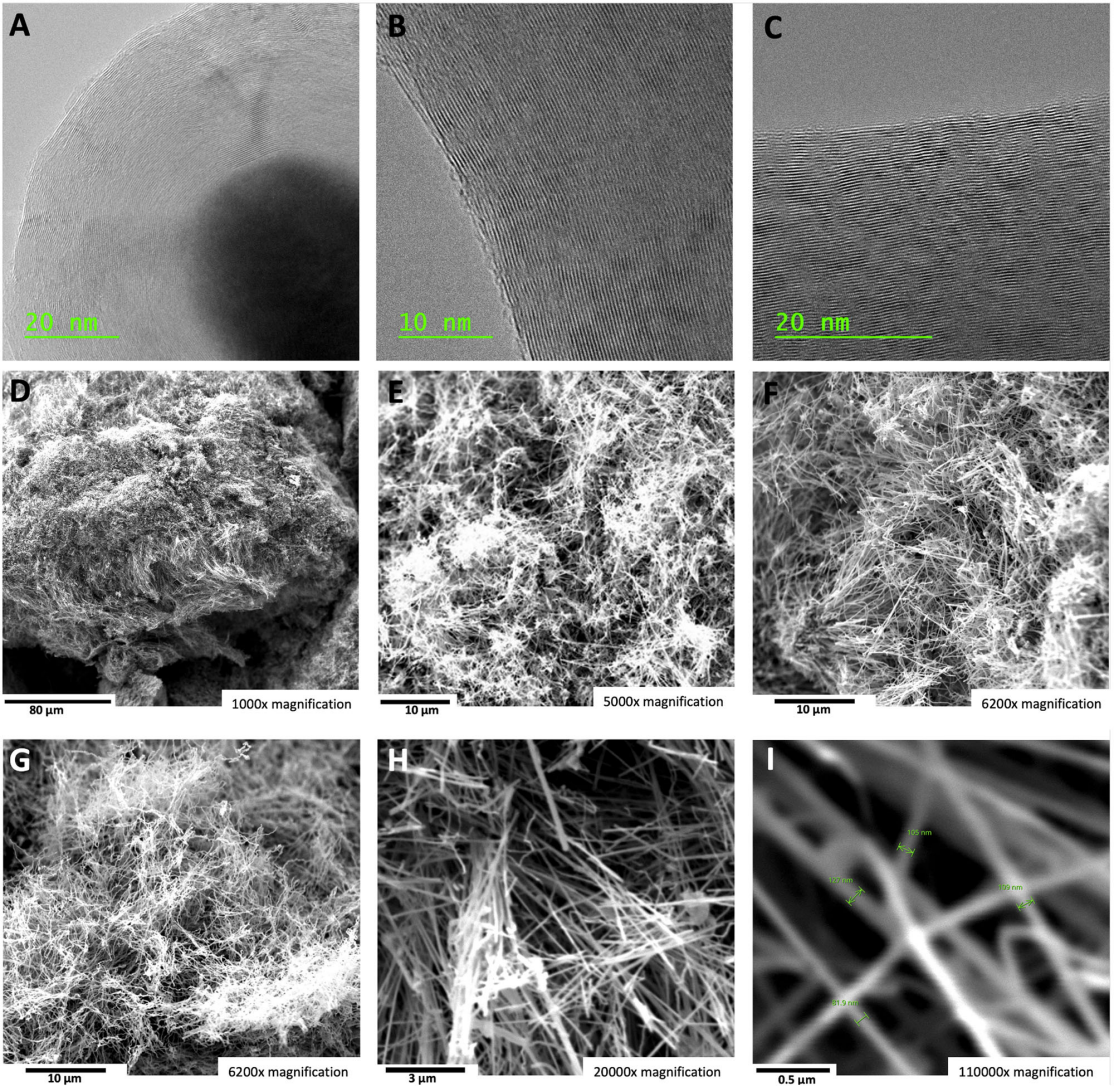
The carbon nanotube product of high current electrolysis in a 45 wt% SrCO_3_ in a Li_2_CO_3_ electrolyte. SEM images of the product from 4-h electrolysis conducted at 790 °C and J = 0.6 A/cm^2^ with a stainless steel 304 anode at a 120 cm^2^ area brass cathode. **A**–**C** TEM with 20 nm (**A** & **C**) and 10 nm (**B**) scale (3.5 to 7 million × magnification) showing the distinctive hemispherical concentric graphene layers surrounding the nucleation catalyst (**C**) and the concentric graphene adjacent layers above and below the hollow core (**B**, **C**). SEM magnification is **D** 1000×, **E** 5000×, **F**, **G** 6200×, **H** 20,000×, or **I** 110,000× magnification.

Figure 6 shows the results obtained for a high concentration (45 wt%) of SrCO_3_ as an electrolyte for electrolysis in the high-current domain of 0.6 A cm^−2^. As seen in TEM (6 A to C) the product remains pure carbon nanotubes graphene walls adjacent to a hollow core. The nucleation metal in the inner tip of the CNT is seen in 6 A. All of the CNTs exhibit a wall of cylindrical graphene layers next to the hollow portion of the CNT as seen in 6B a**n**d 6 C. In 6B, the hollow core and the curving of the cylindrical graphene walls exhibit the typical 0.34 nm separation of graphene layers. Further down the tube, in 6 C are seen the horizontal layered graphene cylinders of the adjacent wall on the other side of the hollow core of the carbon nanotube. We have previously studied the role of the iron, nickel, chromium or other transition metal, individually or in combination, on the formation of various graphene nanocarbon allotropes^4^, or specifically on the carbon nanotube product^1,5,30–33,39^. Initial EDS analysis confirms that iron is the principal nucleating metal for these strontium-based electrolyte carbon nanotubes. A more detailed analysis will be expanded on in a future study.

The 0.6 A cm^−2^ electrolysis domain is pertinent because it provides an industrial high rate of material production. This is the same high current density used in the contemporary high-rate industrial production of aluminum (in which aluminum oxide, rather than carbon dioxide, is electrolyzed) and the current density used in the industrial electrolytic production of magnesium. Electrolysis was conducted at an elevated temperature of 790 °C to enhance mass transport under these higher current density conditions. However, as will be seen in subsequent studies, a lower temperature of 770 °C is also effective under high current density conditions.

As shown in Fig. 6, D through I, SEM at various magnification of the CNT product obtained by high-current density electrolysis in the 45 wt% SrCO_3_ electrolyte yields a comparable quality CNT product to that shown in Figs. 1, 5. We previously found that high current density conditions can induce torsional growth of CNTs^31^, and a minor, but evident, increase in tangling of the carbon nanotubes is observed in the high current density growth product in Fig. 6.

### Electrolyses with 50 and 60 wt% strontium carbon electrolytes

**Fig. 7:**
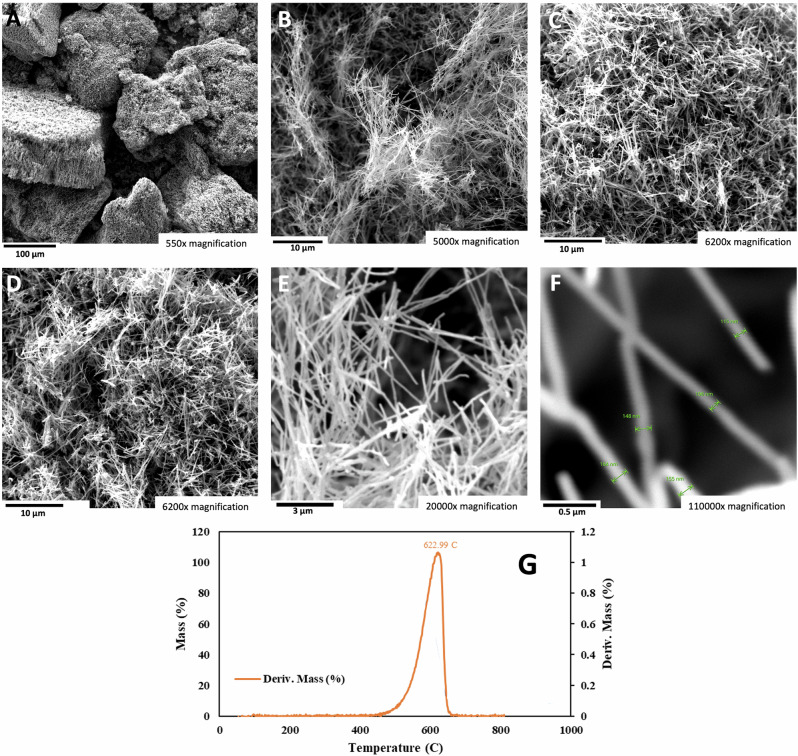
The carbon nanotube product of electrolysis in a 50 wt% SrCO_3_/50 wt% Li_2_CO_3_ electrolyte. SEM images and TGA of the product from 16-h electrolysis conducted at 785 °C and J = 0.28 A/cm^2^ with a stainless steel 304 anode at a 1600 cm^2^ area brass cathode. The SEM magnifications are **A** 15500×, **B** 5000×, **C** 6200×, **D** 6200×, **E** 20,000×, and **F** 110,000× magnification. **G** Differential TGA of the mass loss for the 50% Sr electrolyte product measured with a temperature ramp of 5 °C/minute under air.

During the course of the high-solubility domain experiments summarized in Fig. 3, upon stirring with a stainless steel spatula, the highest-solubility domain (65% SrCO_3_ in Li_2_CO_3_) electrolytes were more viscous. Hence, initial higher domain, 50% SrCO_3_ electrolysis experiments were conducted at higher temperatures and lower than 0.6 A cm^−2^ to overcome the anticipated mass transfer limitations. As shown in the Fig. 7 SEM A through F the product of 785 °C electrolysis at a current density of 0.28 A/cm^2^ continues to be the high-purity CNTs observed as the product of lower SrCO_3_ concentration electrolyses. Due to its lower combustion temperature compared to graphene nanocarbon, amorphous carbon is more susceptible to oxidation, burning easily and exhibiting a TGA derivative of mass versus temperature inflection point, T_infl_, at approximately 300 °C. Alternatively, as seen in Fig. 1, carbon nanotubes possess a high degree of graphitization with typical >600 °C. Similarly, the carbon nanotubes synthesized in the strontium-based electrolytes retain this high degree of graphitization as exemplified for the 50% strontium carbonate electrolyte in Fig. 6G, with measured T_infl_ = 622 °C.

### Strontium electrolyte electrolysis at large electrodes

The results shown in Figs. 5–7 were obtained for small or medium-sized Muntz brass cathodes. Specifically, the electrolyses in Figs. 5, 6 were conducted at cathodes with a surface area under 200 cm^2^, while the Fig. 7 electrolysis utilized a cathode with an area of 1600 cm^2^. Strontium electrolyte electrolysis can be routinely performed with larger cathodes (and at higher current densities) to facilitate large-scale carbon capture.

**Fig. 8:**
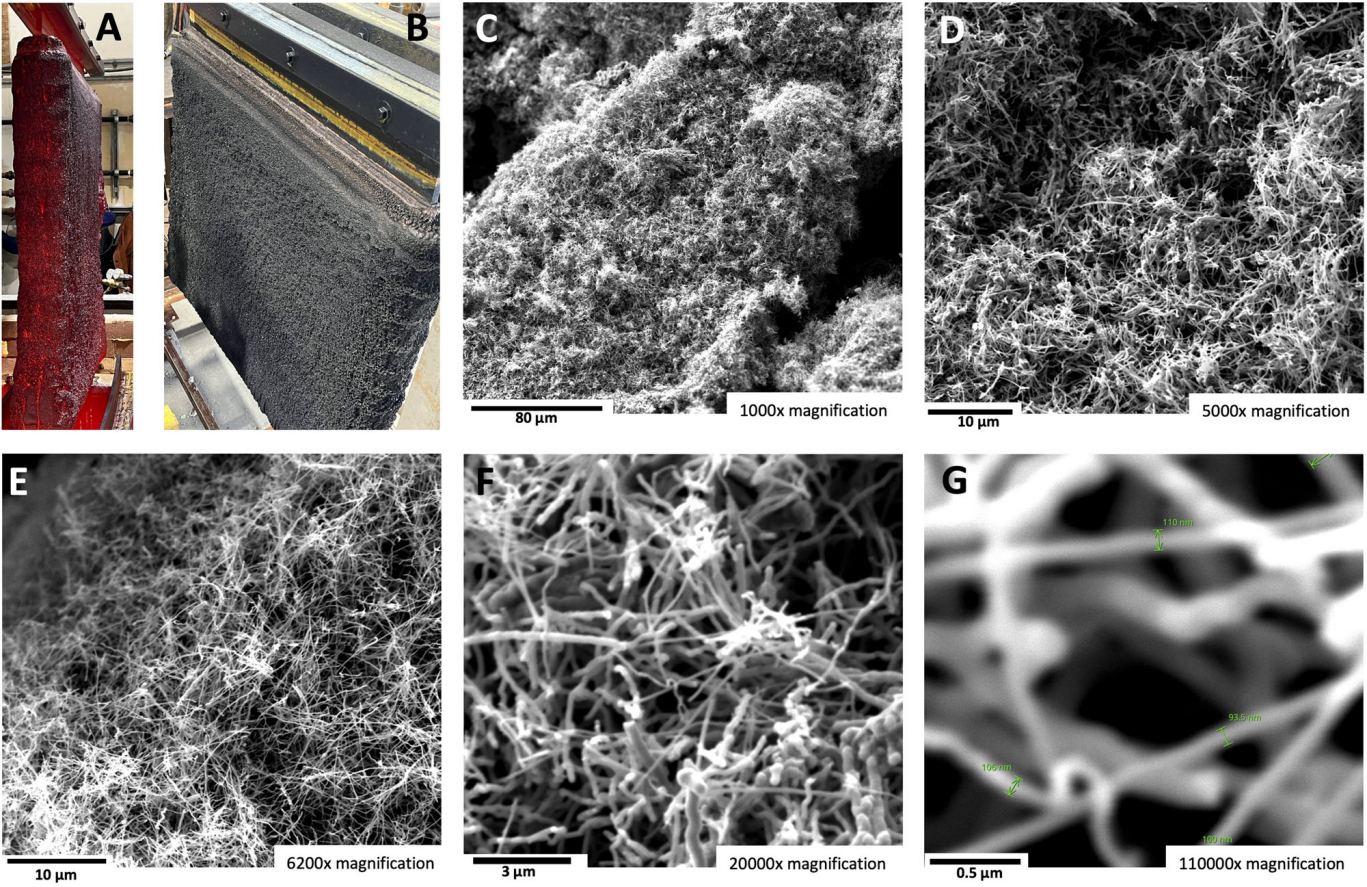
Carbon nanotube product with a high current density, large-area cathode, and 50 wt% SrCO_3_/50 wt% Li_2_CO_3_ electrolyte. **A**, **B** show the cathode, with an active area of 11,000 cm^2^, upon lifting from the electrolyte and subsequent cooling. **A** Electrolysis is conducted at 770 °C and J = 0.6 A/cm^2^ utilizing the stainless steel 304 carbon pot as the anode. The SEM product magnifications are as follows: **C** 1000×, **D** 5000×, **E** 6200×, **F** 20000×, **G** 11000× magnification.

Figure 8 shows the results for a 770 °C 50% SrCO_3_ and 50% Li_2_CO_3_ 0.6 A/cm^2^ current density electrolysis at an 11,000 cm^2^ surface area on a Muntz brass cathode. Panel A of the figure shows the hot Muntz brass cathode subsequent to electrolysis as lifted from the electrolysis chamber below. Panel B shows the same cathode subsequent to cooling. The cathode deposit is approximately 4” thick. SEM characterization of the product of this high surface area, high current density 40% SrCO_3_ electrolysis is shown in the figure. Once again, a high-purity CNT product is obtained, and as was observed in the other high-current density electrolysis in Fig. 6; an increase in the tangling of the carbon nanotubes is evident in the carbon nanotube product.

Note that the cathode in Fig. 8 is vertically oriented. This considerably decreases the physical plant footprint required for C2CNT decarbonization. Aluminum production has been restricted to horizontal electrodes because the aluminum product is molten and, during electrolysis, lies on top of the cathode at the bottom of the aluminum pot. Aluminum can also require greater kiln insulation due to the lower pot packing conditions and aluminum production’s higher 960 °C pot operating temperature.

### 60 to 64% binary and ternary SrCO_3_ electrolytes with 35 to 40% Li_2_CO_3_

**Fig. 9:**
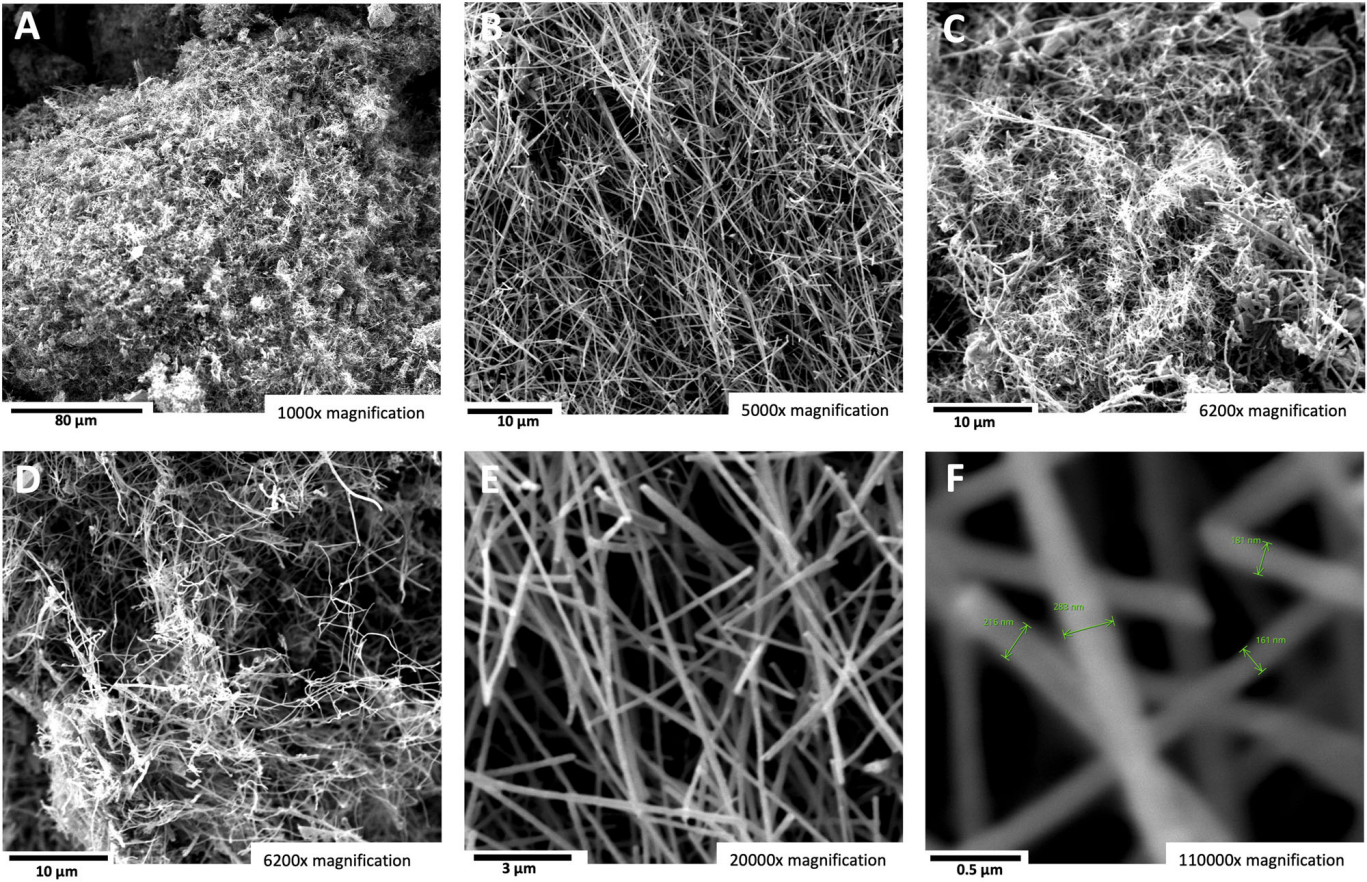
The carbon nanotube product of electrolysis in a 60 wt% SrCO_3_/40 wt% Li_2_CO_3_ electrolyte. SEM images of the product from 16-h electrolysis conducted at 770 °C and J = 0.2 A/cm^2^ with a stainless steel 304 anode at a 288 cm^2^ area brass cathode. The SEM magnifications are **A** 15500×, **B** 5000×, **C** 6200×, **D** 6200×, **E** 20,000×, and **F** 110,000×.

The success of the 50% SrCO_3_ electrolysis suggested that lower temperatures were viable for concentrated electrolytes. Therefore, a 60% SrCO_3_ in 40% Li_2_CO_3_ electrolyte was conducted at 770 °C. Figure 9 summarizes the SEM characterization of the product of the 60% SrCO_3_ electrolysis. Once again, a high-purity CNT product is attained.

We previously observed that low-level Li_2_O additions can improve the purity of Li_2_CO_3_ electrolyses^32^. Oxides can induce twisting of carbon nanotubes due to an increase in sp^3^ defects^1,29,31^ and, in one case, branched rather than discrete CNT forms^4^, and in this case the observed high solubility of strontium oxide adds another component to the electrolyte mix that can decrease the Li_2_CO_3_ component required in the electrolysis. As with Li_2_CO_3_, Li_2_O is an expensive lithium salt due to its scarcity and can be an expensive component to add to molten carbonate electrolytes. As with SrCO_3_, SrO is inexpensive and is an inexpensive additive to molten carbonate electrolytes for decarbonization.

**Fig. 10:**
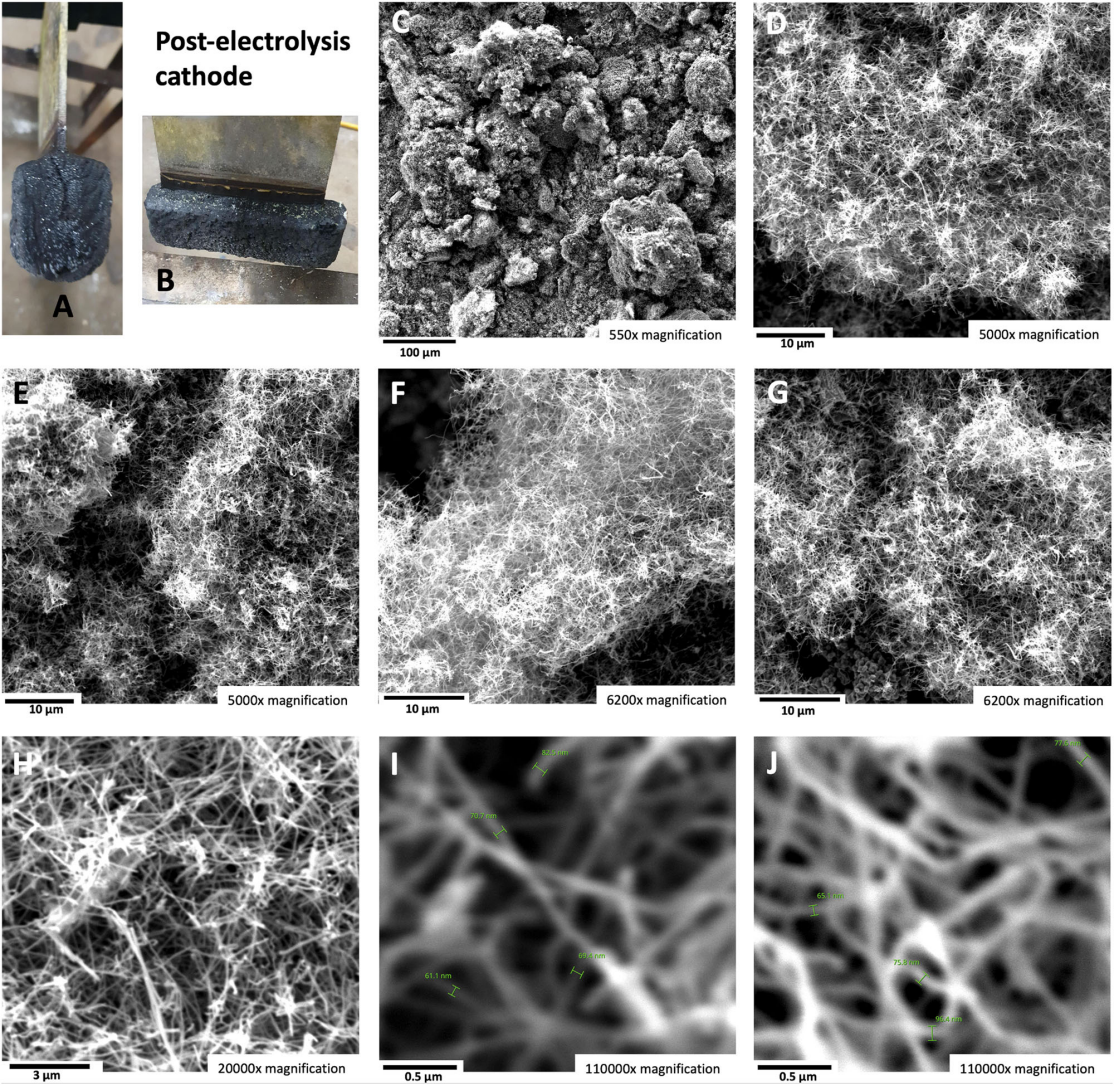
The carbon nanotube product of electrolysis in a ternary 35 wt% Li_2_CO_3_ electrolyte with 64 wt% SrCO_3_ and 1 wt% SrO electrolyte. **A**, **B** Edge and top view of the 2-sided 6 × 8 cm active area Muntz brass cathode with the post-electrolysis cooled product. **C**–**J** SEM images of the product from 4 h of electrolysis conducted at 770 °C and J = 0.6 A/cm^2^ with a stainless steel 304 anode at a 96 cm^2^ area brass cathode. The SEM magnification is **C**: 550×, **D**, **E**: 5000×, **F**, **G** 6200×, **H** 20,000×, or **I**, **J**: 110,000× magnification.

We hypothesize that in the high strontium concentration domain, the addition of strontium oxide as a ternary compound to the binary SrCO_3_/Li_2_CO_3_ system may improve mass transfer by increasing strontium solubility and decreasing viscosity. As shown in Fig. 10, a high-purity CNT product indeed formed at a low Li_2_CO_3_ concentration and high SrCO_3_ concentration upon the addition of SrO as a ternary component. The electrolysis was conducted in a 770 °C electrolyte at a current density of 0.6 A/cm^2^. The 64 wt% SrCO_3_ plus 1% SrO electrolyte contains only 35 wt% Li_2_CO_3_. The product continued to be high-purity CNTs, as had been observed with the lower concentration SrCO_3_ electrolyses. As expected, with the addition of an oxide, the CNTs are more twisted but retain high purity. Interestingly, as shown in panels I and J, the diameter of the carbon nanotubes ranges from 70 to 90 nm, which is smaller than the 100 nm generally observed in electrolytes without added oxides.

### Ternary and quaternary SrCO_3_ electrolytes with boron salts as little as 30% Li_2_CO_3_

We previously demonstrated that the addition of boron as a borate salt to lithium electrolyzed during molten carbonate electrolysis dopes CNTs, increasing the conductivity of the CNT product by an order of magnitude^27,28,33^. Here, the effect of the addition of borax (Na_2_B_4_O_7_•10H_2_O) to a strontium-rich electrolyte on the purity of a carbon nanotube electrolysis product was investigated. Boraxes lose their water at temperatures greater than 602 °C^57^. The electrolytes were probed in 75 wt% Li_2_CO_3_ electrolytes containing either 24/1, 22/3 or 20/5 wt% SrCO_3_/wt% borax. Electrolyses were conducted for 4 or 16 h at 0.6 A/cm^2^ at 800 °C. Each yielded good quality CNTs according to SEM analysis, and their conductivity will be the topic of another study.

**Fig. 11:**
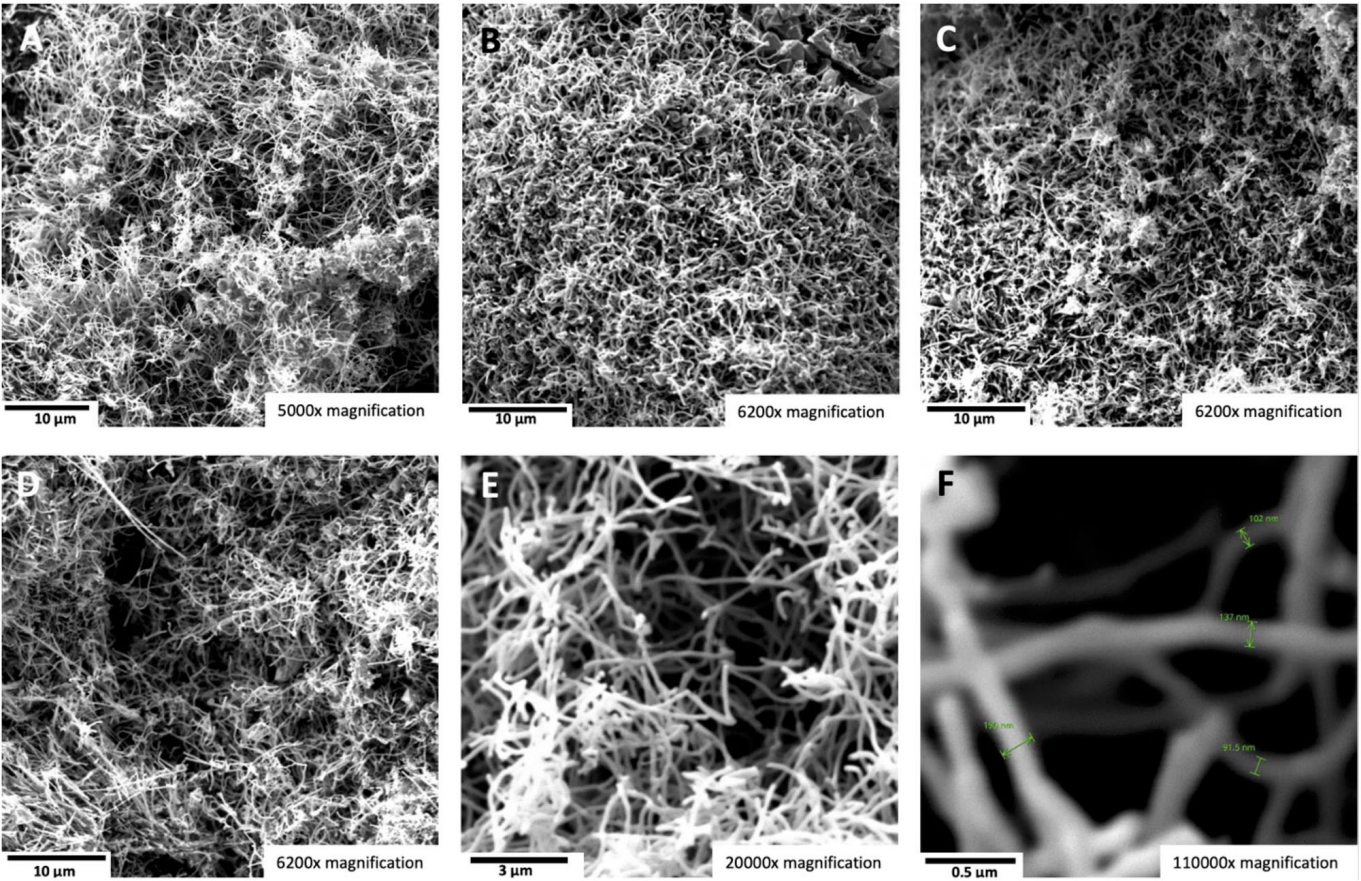
The carbon nanotube product of an electrolysis in a ternary 30 wt% Li_2_CO_3_ electrolyte with 60 wt% SrCO_3_ and 10 wt% SrB_4_O_7_ electrolyte. **A**–**E** SEM of the product from the 4-h electrolysis conducted at 770 °C and J = 0.6 A/cm^2^ with a stainless steel 304 anode at a 96 cm^2^ area brass cathode. The SEM magnification is: **A** 5000×, **B**–**D** 6200×, **E** 20,000×, or **F** 110,000× magnification.

We hypothesize that in the high strontium concentration domain, the addition of strontium borate as a ternary compound to the binary SrCO_3_/Li_2_CO_3_ system may also improve mass transfer by increasing strontium solubility and decreasing the viscosity. In addition to adding another soluble component to the mixture, which tends to decrease the required Li_2_CO_3_ required in electrolyte, in particular, we have observed that borate addition boron dopes and enhances the conductivity of the carbon nanotubes to facilitate the carbon nanotube growth^27,28,33^. SrB_4_O_7_ was synthesized by the reaction of SrCO_3_ + 4H_3_BO_3_ (boric acid), ground together, heat 4 h at 600 °C, reground, then heated overnight at 900 °C forming SrB_4_O_7_ and confirmed by XRD. The electrolysis is conducted in 770 °C electrolyte at a current density of 0.6 A/cm^2^. As seen in Fig. 11, a high-purity CNT product is formed in this low 30 wt% Li_2_CO_3_ concentration, and high 70 wt% Sr salt electrolyte. The product again continues to be the high-purity CNTs observed as those occurring as the product of lower concentration SrCO_3_ electrolyses.

**Fig. 12:**
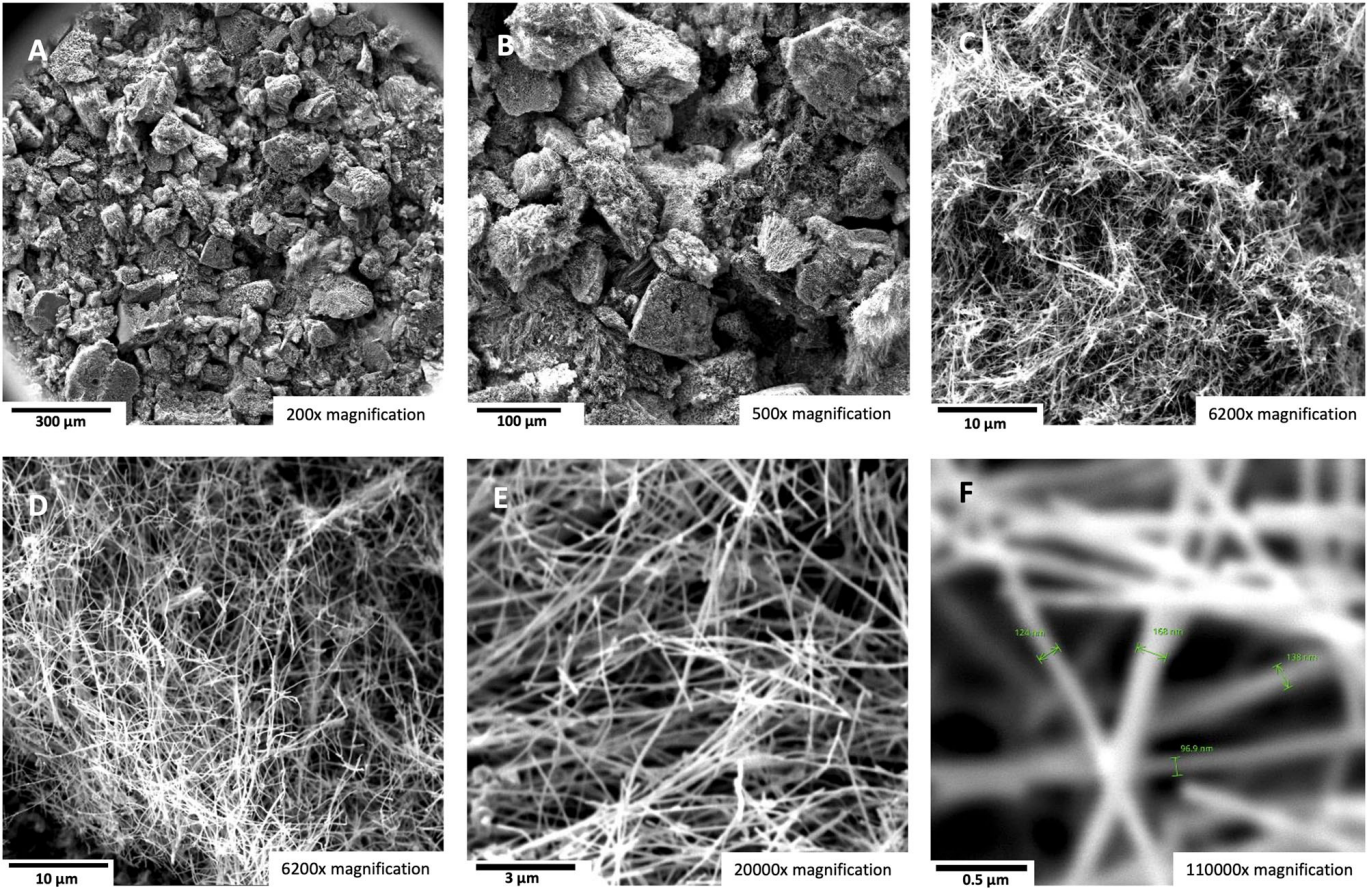
The carbon nanotube product of an electrolysis in a quaternary 30 wt% Li_2_CO_3_ electrolyte with 62 wt% SrCO_3_, 6 wt% B_2_O_3_ and 2 wt% SrO electrolyte. **A**–**E** SEM of the product from the 4-h electrolysis conducted at 770 °C and J = 0.6 A/cm^2^ with a stainless steel 304 anode at a 96 cm^2^ area brass cathode. The SEM magnification is: **A** 200×, **B** 500×, **C**, **D** 6200×, **E** 20,000×, or **F** 110,000× magnification.

A straightforward quaternary SrCO_3_ electrolyte also containing, only 30 wt% Li_2_CO_3_ and boron, leads to the electrosynthesis of high-purity carbon CNTs. Rather than an initial step of the synthesis of SrB_4_O_7_, instead boron oxide (B_2_O_3_ mp 450 °C) was added directly as a component in the electrolyte. Additionally, strontium oxide was added and the solid mix was heated to 800 °C for the electrolysis. Specifically, a 62 wt% SrCO_3_, 6 wt% ^B^_2_^O^_3_ and 2 wt% SrO electrolyte contains only 30 wt% Li_2_CO_3_. Electrolysis was conducted in this electrolyte at 770 °C at current density of 0.6 A/cm^2^ for 4 h. The product of this electrolysis are high-purity CNTs as shown by SEM in Fig. 12.

Ternary and quaternary 30 wt% Li_2_CO_3_ electrolyte generates useful CNTs and are based on a readily available and underutilized SrCO_3_ resource. The electrolytes represent a substantial cost reduction to a major component of the C2CNT decarbonization process.

### A nano-onion product and strontium electrolyte with sodium, barium, or boron salts

**Fig. 13:**
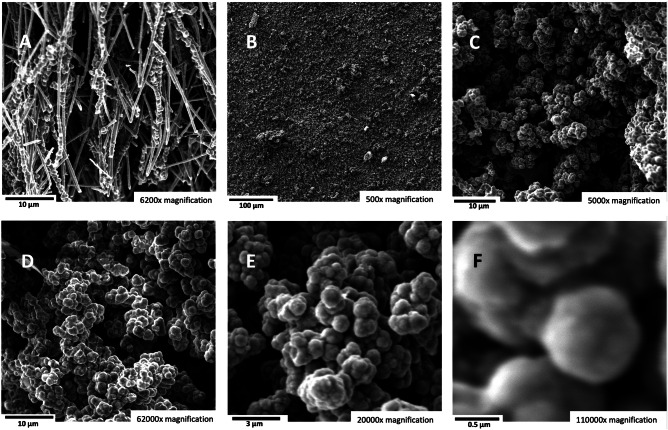
Strontium with sodium carbonate electrolyte produces carbon nano-onions. **A** shows an SEM image of a lower CNO product purity previously grown in the same electrolyte, which shows a mixture of CNTs, CNO, and carbon nanobamboos. Panels (**B**) through (**G**) show the SEM images of a product with a pure CNO product. Electrolyses were conducted in 54 wt% SrCO_3_, 41 wt% Li_2_CO_3_, and 5 wt% Na_2_CO_3_ in an electrolyte at 770 °C and J = 0.6 A/cm^2^ utilizing a stainless steel 304 carbon pot as the anode and a 168 cm^2^ area brass cathode. The SEM magnifications are **A** 6200×, **B** 500×, **C** 5000×, **D** 6200×, **E** 20,000×, and **F** 110,000× magnification.

Interestingly, as shown in Fig. 13, an electrolyte with a low concentration of sodium carbonate can generate a high-purity carbon nano-onion (CNO) product rather than a CNT product. Using a lithium electrolyte without other alkali or alkali earth cations, we previously demonstrated the production of carbon nano-onions, such as in Li_2_CO_3_ containing concentrated (5.9 m) Li_2_O^34^. As shown in the figure, we instead generated carbon nano-onions using a high strontium concentration electrolyte at 770 °C in a 41%/54%/5% lithium carbonate/strontium carbonate/sodium carbonate mixture at 0.6 A/cm^2^. Electrolysis (in the 3rd electrolysis run) generated >90% pure carbon nano-onion products. The percentage of carbon nano-onions in the product increased from 35% CNO after the first electrolysis to 65% CNO after the second electrolysis, to 65% CNO after the third electrolysis still occurred in the same electrolyte and yielded 95% pure CNO at the same electrodes (as shown in Fig. 13 panels B–F). Along with the CNOs, the third electrolysis product contained less than 1% CNTs as shown in panels B–F. A subsequent fourth electrolysis continued to yield similar high-purity CNOs. SEM image of a run of the electrolyte batch that had a lower CNO product is shown in Fig. 13 panel A. The results revealed a mixture of CNTs, CNO, and carbon nanobamboo products, indicating that the growth of the three GNC products was interrelated. The high-purity CNO product was also observed at 770 °C in the 65%/25%/10% lithium/strontium/sodium carbonate mixture at 0.2 A/cm^2^.

The carbon nano-onion product is also generated in the absence of sodium carbonate when electrolysis is perturbed, such as by lowering the cell temperature or by changing the electrolysis anode. For example, when a cell is lined with Nichrome A over the stainless steel of a carbon pot, the anode becomes Nichrome A (composition 80% Ni and 20% chrome), rather than 304 stainless steel, and iron is effectively excluded from the cell. We demonstrated that modifying and inhibiting transition metal nucleation can enhance carbon nano-onion formation^34^. Electrolysis in this Nichrome A anode cell at 770 °C electrolysis in 75%/25% lithium carbonate/strontium carbonate at 0.2 A/cm^2^ produced 90% carbon nano-onions according to SEM inspection.

### Quinary and senary SrCO_3_ electrolytes with as little as 20% Li_2_CO_3_

Sodium carbonate was added as an alternative ternary component to the strontium/lithium binary mixture, for the reason that Na_2_CO_3_ adds an additional high solubility component to the electrolyte^39^, to probe alternative low lithium carbonate domains. A mixture comprising 1/3 of Li_2_CO_3_, 1/3 of SrCO_3_, and 1/3 of Na_2_CO_3_ was fully molten at 750 °C, and at 750 °C, a 4-h electrolysis was conducted at 0.2 A/cm^2^. The CNT product was of good quality and 80–90% purity according to inspection via SEM; although, it was not as high as the >> 90% purity evident in the unitary Li_2_CO_3_ and binary or ternary Li_2_CO_3_/SrCO_3_ electrolytes in Figs. 5–12. However, as expected from the sodium-containing electrolysis potentials in Fig. 2, the electrolysis potential was 0.6 V higher than that of the Na-free electrolytes. The 45 wt% Li_2_CO_3_, 45% SrCO_3_, 9 wt% Na_2_CO_3_ and 1% Li_2_O_2_ concentrations were again higher in voltage, and at 750 °C, 4 h of electrolysis at 0.4 A/cm^2^ resulted in good quality CNT products at 85–90% purity.

A lower concentration of Na_2_CO_3_, along with a high concentration of SrCO_3_ electrolyte, facilitated CNT formation, albeit at a lower quality. This 770 °C electrolysis at 0.6 A/cm^2^ in 50%/45%/5% lithium carbonate/strontium carbonate produced 80% purity CNTs according to SEM inspection. The addition of 1% strontium oxide consistently yielded improved, good-quality 85% purity CNT formation in 770 °C electrolytes at 0.4 or 0.6 A/cm^2^ in 40%/50%/9% or in 40%/54%/5% lithium carbonate/strontium carbonate/sodium carbonate electrolytes containing 1 wt% strontium oxide.

Without added oxide, a 50% strontium carbonate electrolyte containing barium 25% carbonate did not produce CNOs nor CNTs (<10%); this electrolyte was electrolyzed at 770 °C and 25 wt% Li_2_CO_3_/50 wt% SrCO_3_/25 wt% BaCO_3_ at 0.6 A cm^2^. An electrolyte containing both sodium carbonate and barium oxide further increased the electrolysis potential by 0.1 V and generated <50% lower quality purity CNTs at 0.07, 10, 0.20, or 0.40 A/cm^2^ in 40 wt% SrCO_3_, 40 wt% Li_2_CO_3_, 15 wt% Na_2_CO_3_, and 5 wt% BaO electrolyte at 775 °C.

The electrolytic splitting of CO_2_ with electrolytes containing only 30% Li_2_CO_3_, such as the ternary and quaternary electrolyte mixture carbon nanotube products in Figs. 11, 12, achieve the goal in which low availability and expensive Li_2_CO_3_ is no longer a major component of the molten carbonate decarbonization system. A quinary electrolyte that contained only 30% Li_2_CO_3_ and split CO_2_ to produce high-quality carbon nanotubes added strontium chloride to the electrolyte and contained wt% 20/57/5/2/6 of Li_2_CO_3_/SrCO_3_/2% SrO/B_2_O_3_. The synthesis at 800 °C used the same Muntz brass and 304 stainless electrodes and an electrolysis current density of J = 0.6 A/cm^2^ from 770 °C electrolysis in pure Li_2_CO_3_

It is expected, and observed that higher component electrolytes will have the capability to facilitate further dissolution of non-Li components in the molten carbonate decarbonization electrolyte, and thereby to lower lithium carbonate to less than 30. Such extended details should be pursued in further studies. Although this 30% objective has been reached, we will note here that we have further synthesized a high-quality carbon nanotube product with a quinary (5 component) 25% Li_2_CO_3_ 800 °C electrolyte consisting of wt% 25/62/5/2/6 of Li_2_CO_3_/SrCO_3_/Na_2_CO_3_/SrO/B_2_O_3_ under the same 800 °C electrolysis conditions. In another quinary electrolyte producing a good CNT product, we have further decreased the Li_2_CO_3_ content to 20% with an electrolyte of 20/62/10/2/6 of Li_2_CO_3_/SrCO_3_/Na_2_CO_3_/SrO/B_2_O_3_. Finally, in a senary (six component) electrolyte producing a good CNT product under the same electrolysis conditions consists of wt% 20/57/10/2/6/5 Li_2_CO_3_ / SrCO_3_/Na_2_CO_3_/SrCl_2_/SrO/B_2_O_3_.

### Cost analysis of C2CNT decarbonization

This brief analysis draws comparisons with the cost structure of a well-established industry: aluminum production. The C2CNT process shares several characteristics with aluminum smelting. Both involve molten electrolysis and do not require noble or exotic materials. Aluminum smelting converts aluminum oxide into aluminum metal, while C2CNT produces carbon nanotubes from carbon dioxide. Aluminum smelting operates at around 960 °C in a molten cryolite (sodium fluoroaluminate) electrolyte, while C2CNT operates in molten carbonate. Both processes function at high current densities (hundreds of mA per cm²) and exhibit low polarization. The electrolysis chambers in both processes are constructed from common metals, standard insulators (such as kiln or “firebricks”), and control equipment. In aluminum smelting, electrolysis is driven at approximately 4 volts, utilizing 3 electrons per aluminum atom.

A summary of Al production costs per tonne of Al, based on market costs, is presented in Table 1. These costs are averaged from similar values in several studies^58–62^. The $2005 in costs are consistent with today’s market value of $2400 per tonne of aluminum^63^. The costs consist of: Consumable Expenses including materials (52% including alumina, carbon, and cryolite), Electricity: 32%, Labor: 8%, and Capital Expenses (amortized cost of electrolyzers, processing equipment, and miscellaneous overhead). For each tonne of aluminum, the production consumes 5.69 tonnes of alumina (refined bauxite), 0.40 tonne of carbon, and 0.126 tonnes of cryolite^62^. Note that the energy required for aluminum production comes from two sources: electricity and the energy released from the consumed carbon anode.

**Table 1:** Comparison of aluminum and C2CNT production costs and value per tonne product

Process	$US Cost (% of total)							product	Price/tonne
Al smelt	Reactant	carbon	electrolyte	electricity	labor	capital	total		
	alumina 733 (37%)	$211 (12%)	Cryolite $126 (6%)	$602 (30%)	$150 (7%)	$150 (7%)	$2005 (100%)	aluminum metal	$2,400
C2CNT (SrCO_3_)	CO_2_ (0%)	0 (0%)	SrCO_3_ $131 (17%)	$360 (46%)	$150 (19%)	$150 (19%)	$791 (100%)	carbon nanotubes	$40.000–$1,000,000
C2CNT (Li_2_CO_3_)	$0 (0%)	0 (0%)	Li_2_CO_3_ $1950 (75%)	$360 (14%)	$15 (6%)	$150 (6%)	$2610	carbon nanotubes	$40.000–$1,000,000

As shown in Table 1, the C2CNT process differs from aluminum smelting in that it uses a low-cost oxide—carbon dioxide—rather than aluminum oxide (alumina processed from NaOH-treated bauxite). Both processes are straightforward, high-current-density electrochemical methods involving molten electrolytic reduction of oxides. The C2CNT process operates under somewhat milder conditions at approximately 770 °C in a less toxic, molten carbonate electrolyte, and to a first order of approximation, both processes will be assumed to have the same labor costs, tonnage of electrolyte consumption, and capital costs. Whereas, Al production requires ~13 MWh per ton of aluminum at $0.05/kWh, C2CNT production requires less energy (7 MWh) per ton of carbon nanotubes based on the 4-electrons per carbon dioxide splitting. The electrolysis voltage varies from 0.8 V to up to 2 V^1,2^, and an electrical cost of $360 per ton CNT. A major difference in the cost structure is based on electrolyte cost. Whereas costs are ~$1000 per tonne cryolite^64^, and ~$1040 per tonne strontium carbonate^45^, lithium carbonate costs are ~$15,000 per tonne^46^. For the 0.126 tonne of electrolyte, this yields comparative total tonnage costs of $2005 for aluminum, only $791 per tonne for CNTs based on the SrCO_3_ (note high, but not 100%, SrCO_3_ electrolyte was demonstrated in this study), and $2610 per tonne based on the Li_2_CO_3_ electrolyte. These CNT will fluctuate with the large variation in Li_2_CO_3_ cost and the electrolyte waste per tonne of CNT produced (which is assumed here as similar to that of Al production). Note, that the dominant cost in CNT production is the electrolyte and total costs for CO_2_ splitting to carbon nanotubes are over 3-fold higher for the lithium carbonate compared to the new strontium carbonate chemistries. Substantial fluctuations in lithium carbonate due to growing EV demand can further exacerbate this price differential.

The value of carbon nanotubes is considerably higher than that of aluminum or the estimated C2CNT production costs in Table 1. The large price range reflects the different costs of industrial compared to high purity grade carbon nanotubes^65^. Carbon nanotubes have found applications in materials such as medicine, polymers, batteries, cement, and textiles^13–23^. A principal advantage of the C2CNT process is that the graphene nanocarbon products are made from CO_2_. With the larger diameter C2CNT CNTs, there is a greater number of concentric cylindrical walls of graphene. These increased-diameter CNTs exhibit a propensity for higher electrical and thermal conductivity, greater rigidity, enhanced electromagnetic radiation absorption, and better (Li-ion) charge storage. The high electrical storage capacity of C2CNT synthesized CNTs has been demonstrated^29^, their use in strengthened polymers presented^66^, and synthesis procedures for doped, helical, magnetic, thin, thick, tangled, straight, long, and bamboo and pearl morphology hollow core CNTs presented^4,5,27,28,30–33^.

## Conclusions

We have presented in this study a sustainability advance in decarbonization technology to directly address global warming, and removal of the greenhouse gas carbon dioxide. The molten Li_2_CO_3_ transformation of CO_2_ to oxygen and graphene nanocarbons, is a large scale process of CO_2_ removal to mitigate climate change. Sustainability benefits include the stability and storage of the products, and the GNC product value is an incentive for carbon removal. However, the high cost of the Li_2_CO_3_ electrolyte and its competitive use as the primary raw material for EV batteries are obstacles.

Lithium carbonate is less available than strontium carbonate, both due to its lower natural abundance and because of the increasing demand for lithium carbonate for EVs and Li-ion batteries. The high cost of lithium carbonate has been suggested as an impediment to molten carbonate decarbonization by C2CNTs. The carbonate carbonization electrolytes prepared from concentrated strontium carbonate demonstrated in this study are substantially more cost-effective than lithium-based electrolytes. The incompatibility of the high solidus point of SrCO_3_ with the preferred molten carbonate decarbonization range of <800 °C has been overcome by determining that strontium carbonate is unusually soluble (to 65% at 790 °C in lithium carbonate). Ternary or higher carbonate mixed electrolytes can further decrease the lithium concentration in the carbonate electrolyte. The thermodynamic equilibrium for the affinity of strontium carbonate to absorb and release carbon dioxide was calculated and shown to be comparable to that of lithium carbonate and shown to be substantially different from that of the other corresponding alkali and alkali earth carbonate equilibria.

The use of a low-Li electrolyte that can provide an electrolyte melting point within the optimal C2CNT process range for CO_2_ to GNC growth between approximately 700 °C and approximately 800 °C has been investigated for concentrated strontium carbonate electrolytes. The electrochemical potential of molten carbonate electrolysis was investigated, and the results showed that the electrolysis potential is low for both pure lithium and binary strontium/lithium electrolytes but higher for sodium or barium carbonate electrolytes.

Low-lithium electrolysis was performed using a vertical planar Muntz brass, cathode and vertical anodes composed of stainless steel. Effective high-concentration strontium-based electrolytes that produce high-quality GNC products include both binary mixtures (for example, strontium carbonate/lithium carbonate or strontium oxide/lithium carbonate) and ternary mixture electrolytes (for example, strontium carbonate/lithium carbonate/strontium oxide or borate or sodium salts).

A high current density of 0.6 A/cm^2^ is consistent with industrial-rate electrochemical processes. Binary and ternary strontium carbonate electrolytes were systematically probed for CO_2_ electrolytic decarbonization. At 770 °C and a high current density of 0.6 A/cm^2^, 64 wt% SrCO_3_, 35 wt% Li_2_CO_3_, and 1 wt% SrO are among those demonstrated to be effective for high-purity carbon nanotube electrosynthesis and substantially decrease the concentration of lithium carbonate required in the electrolyte. Another concentrated strontium carbonate electrolyte, consisting of 54 wt% SrCO_3_, 41 wt% Li_2_CO_3_, and 1 wt% SrO, is effective for high-purity carbon nano-onion production.

## Methods

### Materials

Lithium carbonate was purchased at a battery grade >99.5% and was used as received. The lithium carbonate had a compositional composition of 99.8% (Li_2_CO_3_, Green Chemical Co.). The strontium carbonate used was 99.4% pure SrCO_3_ (Shendong Zhi Chemical Co. Strontium oxide, SrO (99% purity, Chemsavers) was used as an electrolyte component in this study. A lower purity SrCO_3_ (98.6%; Hengshui Haoye Co.) tested, containing minor ternary mixture components (0.8% BaCO_3_ and 0.2 wt% CaCO_3_) and had comparable solubility to the higher grade SrCO_3_ shown in the solubility section. BaCO_3_ (Alfa Aesar, 99.5%), Na_2_CO_3_ (Alfa Aesar, 99%), Li_2_O (Alfa Aesar, 99.5%), and BaO (Alfa Aesar, 97%) are also combined to form various molten electrolytes.

Muntz brass (0.25 inches thick in <2000 cm^2^ electrolyses and 0.5 inches thick in the larger cathode study) is a high-zinc brass alloy composed of 60% copper and 40% zinc; this material is also referred to as 280 brass. This material serves as the cathode and was purchased from onlinemetals.com and in larger quantities from Marmetal Industries. Electrolysis was conducted in 304 stainless steel “carbon pots”. The pot acts as both the cell case and its inner walls serve as the anode. In one case, as delineated in the text, the inner wall of the pot was lined with Nichrome A to serve as an alternative electrolysis anode.

### Electrolysis and purification

The specific electrolyte compositions are premixed by weight at the noted ratios for each of the electrolytes described. For the electrolysis potential measurements, the electrolyte to be studied was melted at 770 °C in a small (12 cm × 1 × 2 cm × 15 cm tall) 304 stainless steel. A 0.2 cm wide, 1.5 cm long Muntz brass cathode wire was placed 3 mm from a flat, oversized (3 cm × 6 cm) 304 stainless steel anode and immersed in the electrolyte. Electrolysis potentials were measured. Fixed galvanostatic currents were applied, and electrolysis was measured via a DataQ DAQ interface.

**Fig. 14:**
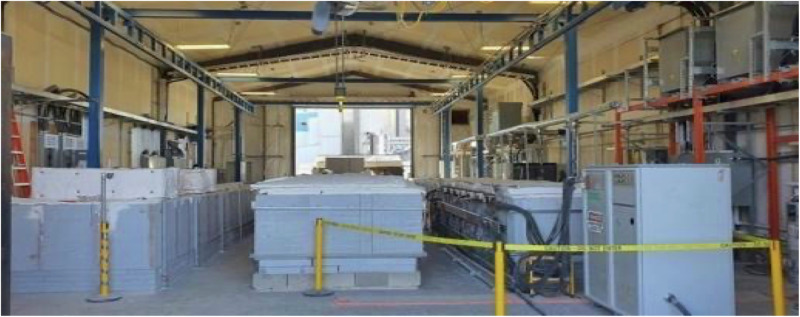
The Genesis Device^®^ kiln used for large-scale CO_2_ molten carbonate electrolysis. The decarbonization kilns are onsite at Carbon Corp in Calgary, Canada.

For the electrolysis experiments, a variety of 304 stainless steels were used. In each case, the cathode is mounted vertically in the electrolyte and across the carbon pot wall, serving as an anode and immersed. Large cathodes, such as those pictured in Figs. 1, 8, are maintained in large thermostatically controlled kilns, as shown in Fig. 14. These kilns simultaneously sustain electrolysis in several carbon pots. The electrolyte has a strong affinity for CO_2_ from the open air, and air was used as the CO_2_ source. The kilns shown in Fig. 14 can also be configured for effective use as an alternative CO_2_ source and direct feed of 5% CO_2_ emissions from the adjacent 860 MW (Shepard, Calgary Canada) natural gas electric power plant. The electrodes are immersed subsequent to electrolyte melt. Once melted, the electrolyte under investigation was maintained at 750 °C to 800 °C, as noted in the text and the figure legends. Electrolysis was conducted galvanostatically with a constant current density. CO_2_ is transformed to carbon, and grows at the cathode as a carbanogel containing a matrix of graphene nanocarbons (GNCs) and a molten electrolyte.

After electrolysis, the raw product from the cathode is cooled, collected from the cathode, ground, remelted, pressed and/or washed with aqueous acid. The washed carbon product was separated by vacuum filtration. The washed carbon product was dried overnight at 60 °C in an oven, yielding a black powder product.

Details of the pressing procedure used to remove excess electrolyte from the product are available^67,68^. This study focused on the optimization of the electrolyte conditions, and for that purpose, a cooled carbanogel containing electrolyte removed from the cathode was retained for use. The hot carbanogel containing molten electrolyte may be pressed directly from the still-hot cathode. This will be shown in subsequent studies.

### Characterization

The carbon products were washed and analyzed by PHENOM Scanning Electron Microscopy and TGA, and is in conjunction with TEM, TEM HAADF, Raman, and XRD, we previously characterized the carbon nanotube products as detailed in the Supplementary Material. In this case, the product purity from 770 °C electrolysis in pure Li_2_CO_3_ is high-purity multiwalled carbon nanotubes. The CNT walls and the CNT hollow cores are evident in panels A and D of supplementary SI Fig. S2. The CNT tip is comprised of carbon, surrounding the nucleation metals of iron with a smaller concentration of nickel in panels C. At higher TEM magnification, the individual, concentric graphene walls are, shown separated by the representative 0.34 nm inter-graphene layer spacing in A-1 panels B1-1, and the pure carbon composition of the CNT cross-section is presented in the bottom righthand panel of Fig. S2. As previously delineated, the CNT diameter and number of graphene walls initially increases with growth time and approaches a limiting diameter^36^. Presumably, the limiting diameter occurs as the nucleating transition metal becomes increasingly buried within the CNT tip. This limit will depend on electrochemical conditions^30^. As one example, after 5, 15, or 90 min of electrolytic growth, the hollow core CNTs grew respectively from 18 to 39 to 142 graphene walls and respectively of 22, 47 to 116 nm diameters in molten 770 °C Li_2_CO_3_^36^. SEM, shown in Fig. 5, were performed with a PHENOM Pro-X SEM (with Energy Dispersive Spectroscopy, EDS), and Figs. 6–11 were measured with a PHENOM Pro-XL High THROUGHPUT SEM. TGA were performed with a Perkin Elmer STA 6000 TGA/DSC instrument with autosampler instrumentation.

## Supplementary information

Supplemental Material

## Data Availability

The source data that support the findings of this study are available from the corresponding authors upon reasonable request. Source data are provided within this paper and in the Supplementary Material.
