# Supplementary material for: A new electrolyte for molten carbonate decarbonization
Source: Commun Chem. 2024 Sep 18;7:211. doi: 10.1038/s42004-024-01306-z (PMC11408528; doi:10.1038/s42004-024-01306-z)
Supplement: Supplementary file 1 — Supplemental Material [file 42004_2024_1306_MOESM1_ESM.pdf]

Supplemental Material accompanying:

A New Electrolyte for Molten Carbonate Decarbonization

Gad Licht, Kyle Hofstetter, Xirui Wang and Stuart Licht
George Washington University, Washington, DC, USA,
Carbon Corp, Calgary, Canada & C2CNT LLC, Florida, USA

Supplementary Discussion

Elevated levels of atmospheric CO_2 are the primary driver of global warming. Historically, atmospheric CO_2 concentration fluctuated within a range of 235 ± ∼50 ppm over the past several hundred thousand years, until 1850. Currently, it stands at 426 ppm and continues to increase annually, leading to widespread climate disturbances, habitat degradation, and species extinction [1-4]. The inherent chemical stability of CO_2 poses a significant challenge to its removal by conversion into a non-greenhouse material, a subject explored in our US NSF workshop on Chemical Recycling and Utilization of CO_2 [5]. However, overcoming this stability of CO_2 and utilizing it as a carbon-negative precursor for the production of valuable products provides an incentive for the reduction of this greenhouse gas.

In 2015, it was shown that the growth of transition metal nuclei during this electrolysis process leads directly to the conversion of CO_2 into pure graphene nanocarbons, including carbon nanofibers and carbon nanotubes (CNTs) [6]. This transformation of the greenhouse gas CO_2 into valuable GNC products offers a chance to convert CO_2 into a form of carbon stabilized by graphene, thus aiding in mitigating climate change. Graphite is an analogous macroscopic form of layered graphene, and as a mineral graphite has an established geologic (hundreds of millions of years) lifetime.

The CO_2 to nanocarbon process, including electrolyte separation and return to the electrolysis chamber, and extraction of the pure GNC product is illustrated Figure S1. As illustrated in Figure 1, during electrolysis CO_2 either sourced directly from the air or industrial emissions are transformed to GNCs by electrolysis in molten carbonates. The CO_2 is split into C and O_2 with a GNC-electrolyte matrix growing at the electrolysis cathode. This nanocarbon/carbonate electrolyte mix has been termed a carbanogel and is refined through the separation of the electrolyte.

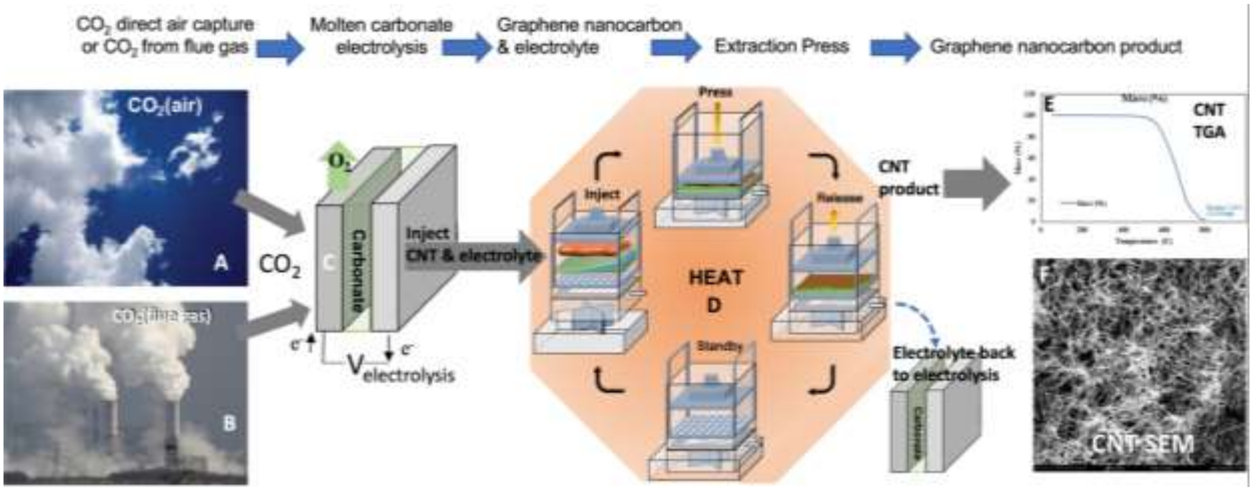

Figure S1. The CO_2 to graphene nanocarbon process. CO_2 sourced either from an anthropogenic source (CCUS) or from the air (DAC), panels A and B, is directly transformed into graphene nanocarbons, panel F. The morphology of the graphene nanocarbon is determined by tuning the electrochemical conditions of the molten carbonate CO_2 electrolysis, panel C. The nanocarbon product (carbon nanotube exemplified) is separated from the molten electrolyte by a high-pressure, high-temperature extraction press, panel D, and has carbon nanotube morphology, as indicated by SEM panel F and in panel E by TGA resistance to high-temperature oxidation as characterized. With parts modified with copyright permission from X. Wang, G. Licht and S. Licht, Green and scalable separation and purification of carbon materials in molten salt by efficient high-temperature press filtration, *Sep. Purif. Technol*., 2021, **244** 117719, DOI: 10.1016/j.seppur.2020.117719.

The high electrical conductivity character of the graphene nano-allotropes supports continuous growth during the CO_2 molten electrolysis at low electrolysis voltage. This cathode product grows as an interconnected matrix with electrolyte in the matrix pores. Deriving its name from aerogels, this matrix containing carbonate electrolyte has been termed a carbanogel. Some of the electrolyte in this matrix is rather loosely bound. For example, a post-electrolysis cathode lifted out of the molten electrolyte can release over 30% of the bound electrolyte by gravitational drip.

Main Figure underlying data, that is TGA in Figures 1, 5 and 7), SEM in Figures 1, and 5-13, Electrolysis Potential and Solubilities measured in Figures 2 and 3 are all included as presented in the the individual Figures. Equilibrium Constants in Figure 4 of the main text are determined as described by Eq 5.

Underlying Data for Figure 1,

		∆G, kJ/mol						
T(K)	T(°C)	**MgCO3**	**CaCO3**	**Li2CO3**	**SrCO3**	**BaCO3**	**Na2CO3**	**K2CO3**
300	**27**	64.8	130.2	175.6	183.0	220.6	274.6	348.1
400	**127**	64.4	129.8	175.3	182.6	220.3	274.3	347.8
500	**227**	47.0	113.9	159.3	165.4	203.0	259.3	332.4
600	**327**	29.7	98.2	143.4	148.4	185.9	244.6	317.0
700	**427**	12.6	82.7	127.8	131.5	169.1	230.2	301.5
800	**527**	-4.2	67.4	112.7	114.7	152.5	216.4	286.2
900	**627**	-20.8	52.3	98.4	98.0	136.1	203.0	271.0
1000	**727**	-37.1	37.4	84.4	81.5	120.0	189.9	256.0
1100	**827**	-53.2	22.6	71.2	71.5	104.2	177.0	241.1
1200	**927**		8.1	62.6	55.3	89.0	164.3	226.5
1300	**1027**		-6.4	54.4	39.3	75.5	153.8	212.7
1400	**1127**		-20.6	46.5	24.9	62.6	143.8	200.9
1500	**1227**		-34.7	38.8	10.7	50.1	133.6	189.3
1600	**1327**		-48.7	31.4	-3.3	38.0	120.3	177.9

Control of the electrode and electrolyte composition, and CO_2 electrolysis splitting temperature and current density tunes the decarbonization process to form a range of high purity graphene nanocarbon products, including carbon nanotubes. Typical SEM, TEM and HAADF (High Angle Annular Dark-Field TEM) elemental analysis imaging of the CNTs are presented in Figure S2, and have been extensively detailed [7].

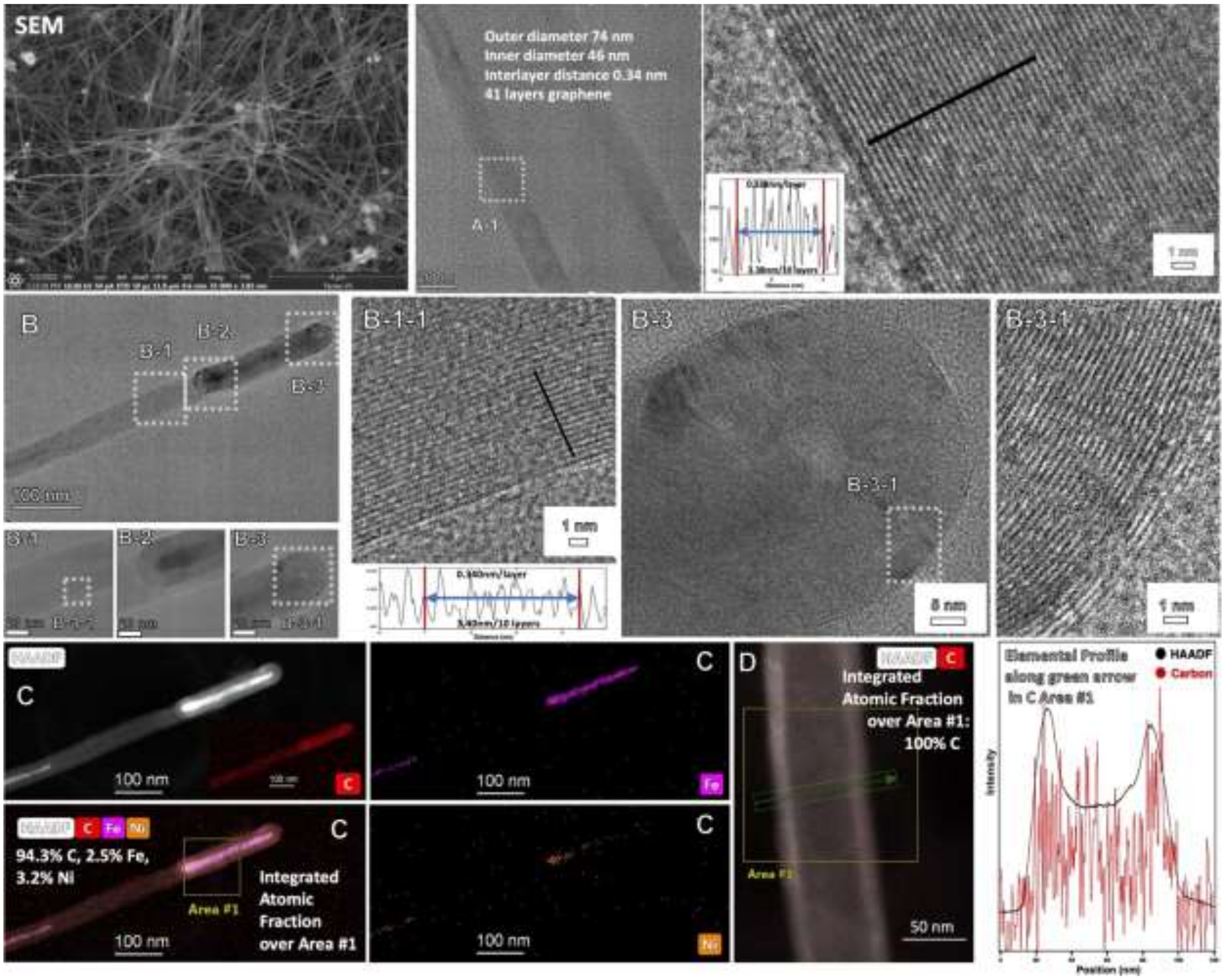

Figure S2. SEM TEM and HAADF of the synthesis product of high purity, high yield carbon nanotubes by electrolytic splitting of CO_2 in 770°C Li_2CO_3. The SEM has a scale bar of 5 µm. Panels B are TEM with scale bars decreasing from 100, 20 nm, 5 and 1 nm. Bottom rows panels C are HAADF elemental analyses with scale bars decreasing from 100 to 50 nm, and in the bottom right a HAADF elemental carbon profile analysis of the carbon nanotube cross section. in the bottom right a HAADF elemental carbon profile analysis of the carbon nanotube cross section. Modified from open access paper X. Liu, G. Licht, S. Licht, Controlled Transition Metal Nucleated Growth of Carbon Nanotubes by Molten Electrolysis of CO_2 Catalysts 12 (2022) 137. https://doi.org/10.3390/catal12020137.

Control of the CO_2 electrolysis conditions is used to tune the specific GNC generated by control of the temperature, current density, and the composition of the electrolyte [8]. For example, a lower temperature (725°C) is typically used in the electrolytic growth of carbon nano-onions, while higher temperature (750 to 770°C) is used in the electrolytic growth of carbon nanotubes. Lithium carbonate, a typical electrolyte, has a melting point of 723°C. Binary lithium carbonate mixtures have a lower melting point. A high sodium carbonate content in a mixed sodium/lithium carbonate electrolyte and a lower electrolysis temperature (670°C) drive

the formation of a graphene scaffold nanocarbon product formation. Applied electrolysis current densities generally range from 0.03 to 0.6 A cm^{-2}. High current density (0.6 A cm^{-2} or over) is one of the principal conditions driving the formation of fascinating helical, rather than straight, carbon nanotubes.

Electrode (and electrolyte additive) composition variation has been used to grow a number of other GNC allotropes from CO_2. These include carbon nanobamboo, carbon nanopearl, graphene from nanocarbon platelets, carbon nanofiber, carbon nanobelt, carbon nanotree, and other specific carbon allotrope morphologies. SEM of a range of these GNC products is presented in Fig. S3, and XRD and Raman spectra of the products are presented in Figs. S4 and S5 as previously detailed [8].The solid graphene nanocarbon product from CO_2 grows as a matrix directly on the cathode. Under constant current electrolysis conditions, the product formation is continuous, and the growth occurs in the direction towards the anode.

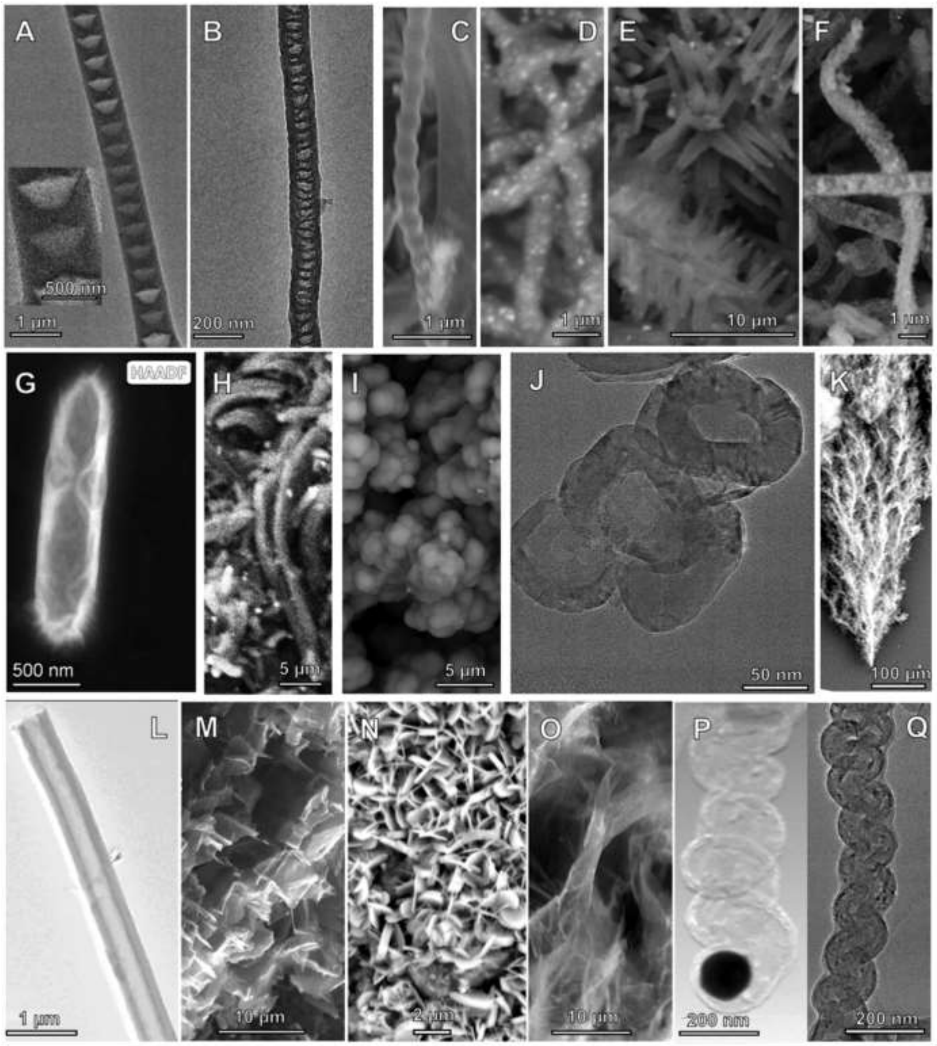

Figure S3. SEM of nanocarbon allotropes synthesized by the electrolytic splitting of CO_2 in molten carbonate. Top row (from A to F) conical CNF, nano-bamboo, nano-pearl, Ni coated CNT, nano-flower, nano-dragon. Middle row (from G to K): nano-rod, nano-belt, nano-onion, hollow nano-onion, and nano-tree. Bottom row (from L to Q) Carbon nanotube, nano-scaffold (ref. 50), nano-platelet, graphene, nano-helices. Bottom row (from L to Q) Carbon nanotube, nano-scaffold (ref. 50), nano-platelet, graphene, nano-helices. Modified from open access paper X. Liu, G. Licht, X. Wang, S. Licht, Controlled Growth of Unusual Nanocarbon Allotropes by Molten Electrolysis of CO_2. Catalysts 12 (2022) 137. https://doi.org/10.3390/catal12020125.

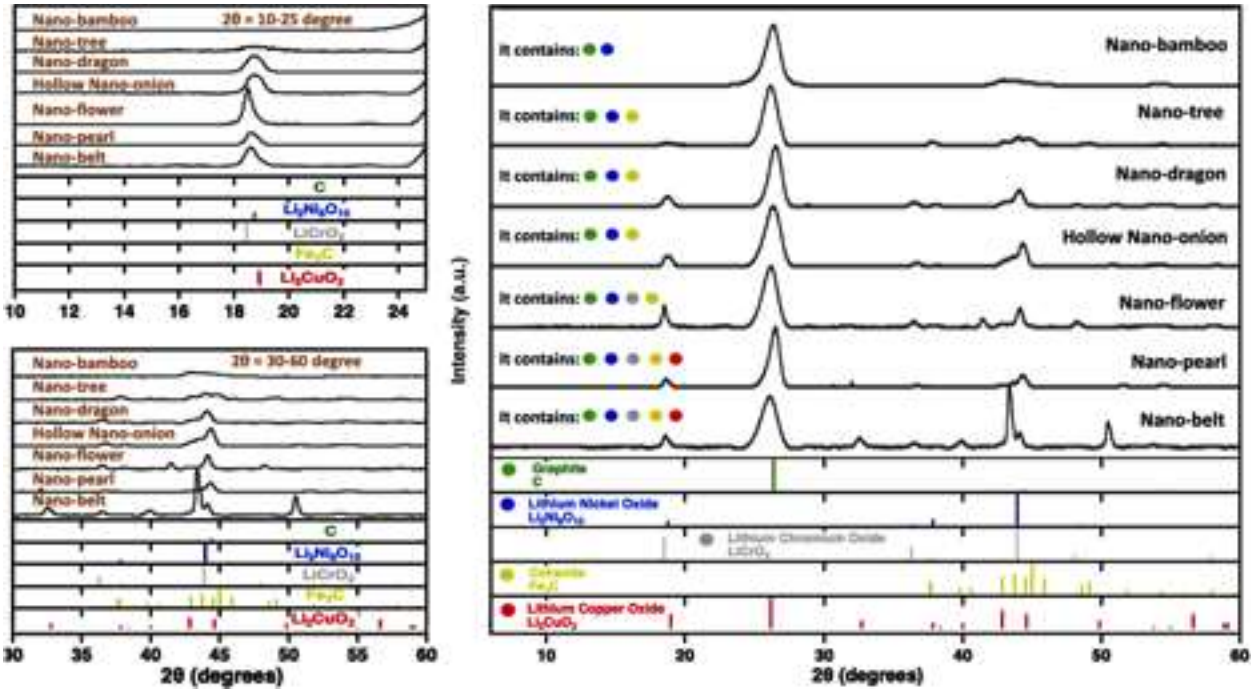

Figure S4. XRD of the synthesis product consisting of various labeled unusual nanocarbon morphologies synthesized by the electrolytic splitting of CO_2 in 770°C Li_2CO_3 with a variety of systematically varied electrochemical conditions. Bottom row (from L to Q) Carbon nanotube, nano-scaffold (ref. 50), nano-platelet, graphene, nano-helices. Modified from open access paper X. Liu, G. Licht, X. Wang, S. Licht, Controlled Growth of Unusual Nanocarbon Allotropes by Molten Electrolysis of CO_2. Catalysts 12 (2022) 137. https://doi.org/10.3390/catal12020125.

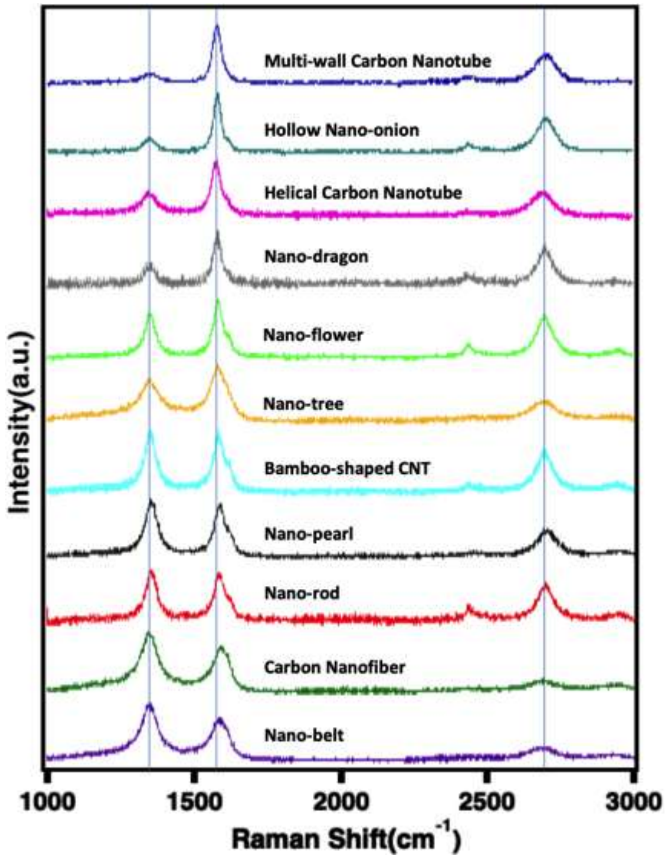

Figure S5. Raman of the synthesis product consists of various labeled GNCs and packed carbon nanotube assemblies synthesized by the electrolytic splitting of CO_2 in 770°C Li_2CO_3 with a variety of systematically varied electrochemical conditions. Bottom row (from L to Q) Carbon nanotube, nano-scaffold (ref. 50), nano-platelet, graphene, nano-helices. Modified from open access paper X. Liu, G. Licht, X. Wang, S. Licht, Controlled Growth of Unusual Nanocarbon Allotropes by Molten Electrolysis of CO_2. Catalysts 12 (2022) 137. https://doi.org/10.3390/catal12020125.

Figure S6 illustrates larger vertical presses that have been scaled up, which include the transfer of applied pressure to the pressing chamber using a hydraulic ram, as described previously[9]. In Figure S6A, there's a cross-sectional depiction of an intermediate scaled-up carbanogel electrolyte extraction unit, detailing the plunger, filter screen platform, and electrolyte exit chamber [9]. Figure S6B shows a larger carbanogel electrolyte extraction unit in operation, capable of pressing up to 0.25 tonnes of carbanogel. Presses in the unit with 50 kg carbanogel have already achieved over 99% electrolyte extraction efficiency.

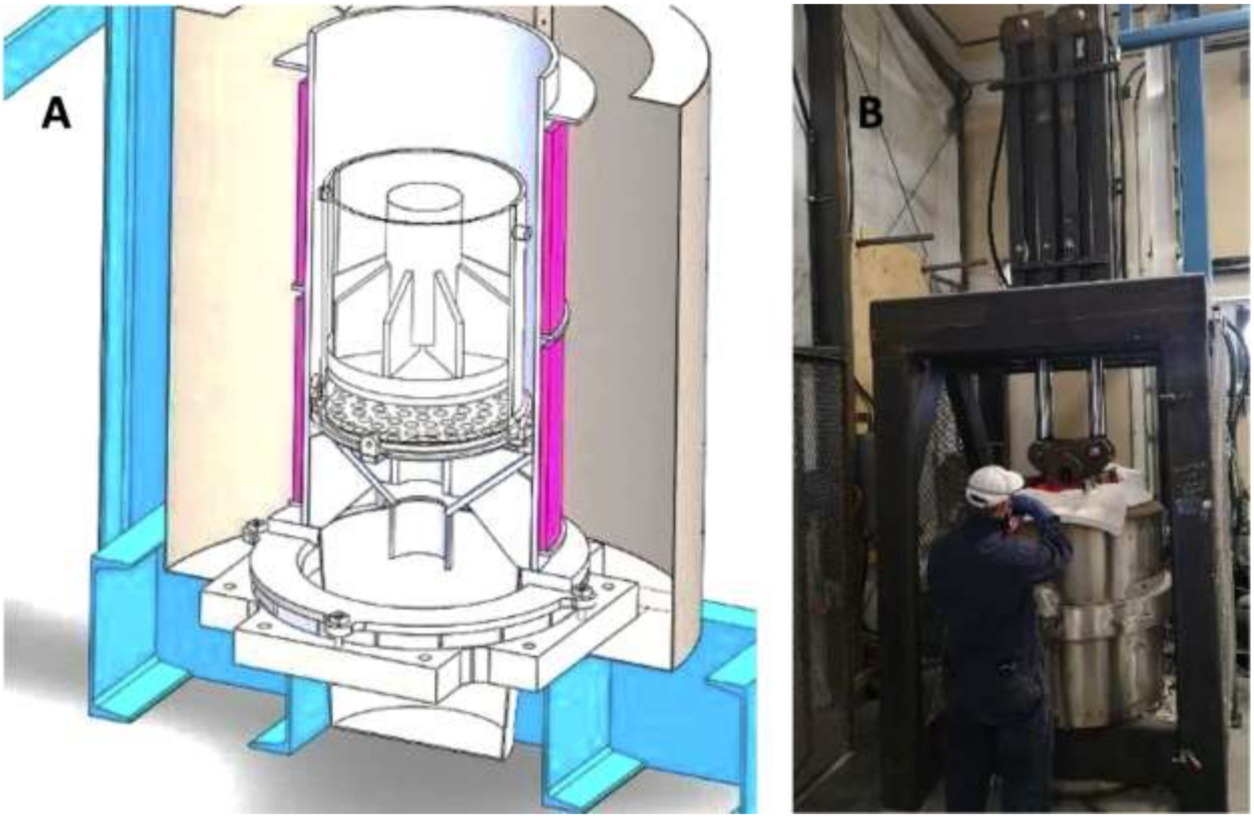

Figure S6. Scaled up carbanogel electrolyte extraction units. A) Cross-sectional illustration of a mid-scaled extraction unit illustrating the plunger, the filter screen platform and the electrolyte exit chamber. B) Larger scaled carbanogel electrolyte extraction unit in action. Licht, K. Hofstetter, S. Licht, Separation of molten electrolyte from the graphene nanocarbon product subsequent to electrolytic CO_2 capture. Decarbon 4 (2024) 100044. https://doi.org/10.1016/j.decarb.2024.100044.

Supplementary References

[1] CO_2-Earth: Daily CO_2 Values. Available online: https://www.CO2.earth/daily-CO2 (accessed on 6 June 2024).

[2] NASA: Global Climate Change: The Relentless Rise of Carbon Dioxide. Available online: https://climate.nasa.gov/climate_ resources/24/ (accessed 6 June 2024).

[3] M. C. Urban, Accelerating extinction risk from climate change, Science 348 (2015) 571–573. https://www.science.org/doi/10.1126/science.aaa4984.

[4] S. L. Pimm, Climate disruption and biodiversity, Curr. Biol. 19 (2009) R595–R601. https://doi.org/10.1016/j.cub.2009.05.055.

[5] G. K. Praksh, G. A. Olah, S. Licht, N. B. Jackson, Reversing Global Warming: Chemical Recycling and Utilization of CO_2; Report of 2008 NSF Workshop; University of Southern California: Los Angeles, CA, USA, 2008. https://www.nsf.gov/mps/che/workshops/2008_ReversingGlobalWarming_NSF_Workshop.pdf (accessed 6 June 2024).

[6] J. Ren, F.-F. Li, J. Lau, L. Gonzalez-Urbina,S. Licht, One-pot synthesis of carbon nanofibers from CO2. Nano Lett. 15 (2015) 6142–6148. DOI: 10.1021/jp9044644.

[7] X. Liu, G. Licht, S. Licht, Controlled Transition Metal Nucleated Growth of Carbon Nanotubes by Molten Electrolysis of CO_2 Catalysts 12 (2022) 137. https://doi.org/10.3390/catal12020137.

[8] X. Liu, G. Licht, X. Wang, S. Licht, Controlled Growth of Unusual Nanocarbon Allotropes by Molten Electrolysis of CO_2. Catalysts 12 (2022) 137. https://doi.org/10.3390/catal12020125.

[9] G. Licht, K. Hofstetter, S. Licht, Separation of molten electrolyte from the graphene nanocarbon product subsequent to electrolytic CO_2 capture. Decarbon 4 (2024) 100044. https://doi.org/10.1016/j.decarb.2024.100044.
